# Minimally invasive versus burr hole craniostomy for chronic subdural haematoma evacuation: a systematic review and meta-analysis

**DOI:** 10.1007/s00701-026-06912-5

**Published:** 2026-05-22

**Authors:** Shaan Patel, Shiva A. Nischal, Angelette Mendonca, Kush M. Kale, Santosh Guru, Pious D. Patel, Kevin Hines, Jack Jallo, Srinivas K. Prasad

**Affiliations:** 1 Department of Neurological Surgery, Thomas Jefferson University Hospital, Philadelphia, PA USA; 2 Department of Physiology, Anatomy & Genetics, Medical Sciences Division, University of Oxford, Oxford, UK; 3 School of Clinical Medicine, University of Cambridge, Cambridge, UK

**Keywords:** Twist-drill, Burr hole, Minimally invasive, Craniotomy, Subdural hematoma

## Abstract

**Background:**

Minimally invasive surgery (MIS) has emerged as an appealing alternative to burr hole craniostomy (BHC) for chronic subdural haematoma (cSDH), offering reduced operative trauma and anaesthetic exposure. However, whether these procedural advantages translate into comparable outcomes remains uncertain. This systematic review and meta-analysis aimed to compare the safety, effectiveness, and procedural performance of MIS versus BHC for cSDH evacuation, with attention to device-specific outcomes.

**Methods:**

A systematic search of PubMed, Embase, and CENTRAL (10th November 2025) identified randomised and observational studies directly comparing MIS techniques, including twist-drill craniostomy (TDC), subdural evacuating port systems (SEPS), and YL-1 devices, with BHC. Primary outcomes were reoperation, recurrence, and overall complications. Secondary outcomes included mortality, clinical, and functional recovery, operative time (OT), and length of hospital stay (LOS). Random-effects meta-analysis using REML generated pooled risks ratios (RRs) and mean differences (MDs). Quality and risk of bias was assessed using RoB 2, ROBINS-I, and GRADE.

**Results:**

Twenty-seven studies encompassing 3752 patients (MIS 1763; BHC 1989) met inclusion criteria. MIS was associated with significantly higher reoperation risk (18.3% versus 10.2%; RR 1.57; 95% CI 1.17–2.10; *P* < 0.001) and higher recurrence in SEPS-specific analyses (RR 1.73; 95% CI 1.29–2.32). Overall complications were lower with MIS (RR 0.63, 95% CI 0.42–0.94; *P* < 0.05). Mortality and postoperative functional outcomes were comparable between groups. MIS substantially reduced OT (MD -24.5 min; *P* < 0.001) and LOS (MD -1.97 days; *P* < 0.01). Device-level heterogeneity was marked: SEPS demonstrated poorest durability, whereas YL-1 systems showed lowest complication risk. RCT-specific subgroup analyses demonstrated directional consistency with the main effect for all primary outcomes. Risk of bias was moderate to serious for most included studies, with moderate certainty of evidence.

**Conclusions:**

MIS offers meaningful perioperative advantages but at the cost of reduced durability, particularly for SEPS-based drainage. BHC remains the more definitive intervention for cSDH, although MIS retains an important role in carefully selected patents, particularly where minimising procedural burden or perioperative morbidity is prioritised. Device-specific performance and haematoma morphology should guide surgical strategy as the field moves toward more mechanistically informed, patient-centred care.

**Supplementary Information:**

The online version contains supplementary material available at 10.1007/s00701-026-06912-5.

## Introduction

Chronic subdural haematoma (cSDH) represents one of the most rapidly expanding neurosurgical burdens in ageing populations, driven by increasing longevity, cerebral atrophy, and widespread antithrombotic exposure [3, 7, 54]. Contemporary epidemiological studies indicate an incidence approaching 20 per 100,000 person-years, with a disproportionate rise among adults over 80 years, positioning cSDH to become one of the most common neurosurgical diagnoses in high-income health systems in the next decade [31, 36] Although traditionally conceptualised as a passive collection of blood products following minor trauma, cSDH is now recognised as a dynamic, self-propagating disorder in which inflammatory neomembranes, fragile neovasculature, and persistent fibrinolysis sustain recurrent microhaemorrhage and proteinaceous exudation [5]. This pathological milieu, rather than the inciting bleed alone, drives progressive enlargement, mass effect, neurological decline, and the significant rates of recurrence seen after initial treatment [10, 17, 55].

Despite advances in perioperative care, recurrence following seemingly successful evacuation remains common, affecting approximately 10–20% of patients and frequently necessitating reoperation. Importantly, recurrent cSDH is not benign: patients requiring repeat intervention face higher risks of seizure, empyema, infection, and further recurrence, as well as prolonged hospitalisation, delayed rehabilitation, and increased healthcare expenditure [36, 37, 46].

Burr hole craniostomy (BHC) with closed-system drainage remains a commonly used operative approach, offering robust decompression through generous access for irrigation, membrane disruption, and evacuation of septated or organised components. However, minimally invasive surgery (MIS), including twist-drill craniostomy (TDC), subdural evacuating port systems (SEPS), and other percutaneous drainage devices (such as YL-1 puncture needle), have gained prominence as clinicians seek strategies that reduce operative trauma, avoid general anaesthesia, and streamline patient flow [3, 11, 25]. These techniques are attractive in frail older adults and in systems under capacity pressure, offering potential advantages in operative time (OT), perioperative morbidity, and length of hospital stay (LOS).

Despite this appeal, the durability of MIS remains debated. Reoperation remains a key concern, with device-specific factors such as small-calibre fenestrations, limited irrigation, and restricted access to organised clot potentially compromising the completeness of evacuation. Conversely, MIS platforms may mitigate air entrainment, wound complications, and anaesthetic exposure, producing a genuinely distinct perioperative risk profile relative to BHC. Importantly, while recent randomised-controlled trials (RCTs) have evaluated the adjunctive use of middle meningeal artery (MMA) embolisation, its role remains evolving and incompletely defined, and these studies do not resolve the foundational question of which primary drainage strategy should be preferred at the index intervention, with heterogenous results across trials [22, 43].

Multiple systematic reviews and meta-analyses have compared TDC, BHC, and other drainage strategies, but heterogeneity in device groupings, drainage protocols, and outcome definitions has limited clear guidance on the relative merits of newer MIS platforms versus conventional BHC and made it difficult to delineate the true trade-offs between efficiency, safety, and durability [23, 26, 35, 59]. Against this backdrop, a rigorous synthesis of the comparative performance of MIS versus BHC is urgently needed. The present systematic review and meta-analysis evaluates these approaches and focusses on reoperation, recurrence, complications, operative efficiency, LOS, mortality, and functional outcomes. By analysing device-specific subgroups, methodological quality, and sources of heterogeneity, this study seeks to clarify where MIS offers true clinical value, where its limitations remain, and how these findings should inform rational, patient-centred surgical decision-making.

## Materials and methods

### Study design and reporting framework

This systematic review and meta-analysis was conducted in accordance with Preferred Reporting Items for Systematic Reviews and Meta-Analyses (PRISMA) guidelines and AMSTAR-2 methodological standards. A protocol was registered a priori on the Prospective Register of Systematic Reviews (PROSPERO) (ID CRD420251242688). AMSTAR-2 criteria were additionally used to benchmark the quality of existing systematic reviews in this field.

### Search strategy and study selection

A comprehensive electronic search of PubMed, Embase, and CENTRAL was performed on 10th November 2025. The search strategy (Supplementary Table 1) incorporated controlled vocabulary and free-text terms for cSDH and MIS drainage techniques, including TDC, SEPS, YL-1 devices, hollow screws, and other percutaneous approaches, as well as BHC. Searches were restricted to English-language articles. Reference lists of included studies and prior systematic reviews were manually screened to identify eligible studies.

Eligible studies were randomised or observational studies enrolling adults (≥ 18 years) and directly comparing MIS drainage techniques with BHC while reporting at least one relevant clinical outcome. Exclusion criteria included any use of middle meningeal artery embolisation (MMAE) (whether performed preoperatively, postoperatively, or concurrently with surgical evacuation), case reports, case series, review articles, conference abstracts, cadaveric studies, studies with no extractable outcome data, and non-English languages studies without translation.

Primary outcomes included: (i) reoperation (defined as any unplanned repeat surgical procedure for ipsilateral cSDH during index admission or follow-up) (ii) recurrence (defined as radiologically confirmed reaccumulation or interval expansion of the ipsilateral cSDH after the index procedure); overall complications (composite outcome defined as any reported systemic or procedure-related adverse event temporally related to the index procedure). Secondary outcomes included mortality, complete clinical resolution defined as resolution or near-complete resolution of presenting neurological symptoms as reported by the original study authors irrespective of radiographic findings, functional outcome via postoperative Markwalder Grading Scale (MGS) score, postoperative intracranial bleeding, postoperative pneumocephalus, postoperative infection, postoperative seizure, OT, LOS.

Title-and-abstract screening was performed independently by two reviewers using Rayyan [32], followed by full-text assessment for inclusion. Disagreements were resolved by consensus with a third reviewer. Inter-rater reliability was calculated using Cohen’s kappa score (κ = 0.82), indicating almost perfect agreement.

### Data extraction and quality assessment

Data was independently extracted by two reviewers, including study design, country, sample size, demographics, and all predefined outcomes of interest stratified by intervention group. Certainty of evidence for each outcome was assessed using the GRADE framework [41]. Risk of bias was evaluated using Cochrane’s Risk-of-Bias tool 2 (RoB 2) for RCTs [47] and Risk of Bias in Non-Randomised Studies of—Interventions tool (ROBINS-I) for non-randomised studies [48], with disagreements adjudicated by a third reviewer.

### Statistical analysis

In accordance with the pre-specified analysis plan, and anticipating moderate-to-high heterogeneity, random effects meta-analysis using the Restricted Maximum-Likelihood (REML) method was performed for outcomes with sufficient data. Dichotomous outcomes were summarised as risk ratios (RRs) and continuous outcomes as mean differences (MDs), each with corresponding 95% confidence intervals (CIs). Statistical heterogeneity was assessed using Cochran’s Q test (*P* < 0.05 indicating significant heterogeneity) and I^2^ statistics (> 25% considered meaningful). A significance threshold of α < 0.05 was applied.

Leave-one-out sensitivity analyses were conducted for all statistically significant outcomes to evaluate the robustness of pooled estimates. Prespecified subgroup analyses were conducted according to MIS device type (TDC, SEPS, YL-1 puncture needle) with each device group analysed separately against BHC to explore device-level differences in outcomes. To mitigate confounding by indication inherent to observational comparisons, and additional prespecified subgroup analysis restricted to RCTs was performed for primary outcomes. Publication bias was assessed using funnel plots for analyses with ≥ 10 studies (limited power below this threshold. All analyses were performed using Review Manager (RevMan) version 9.7.0 [38].

## Results

**Fig. 1:**
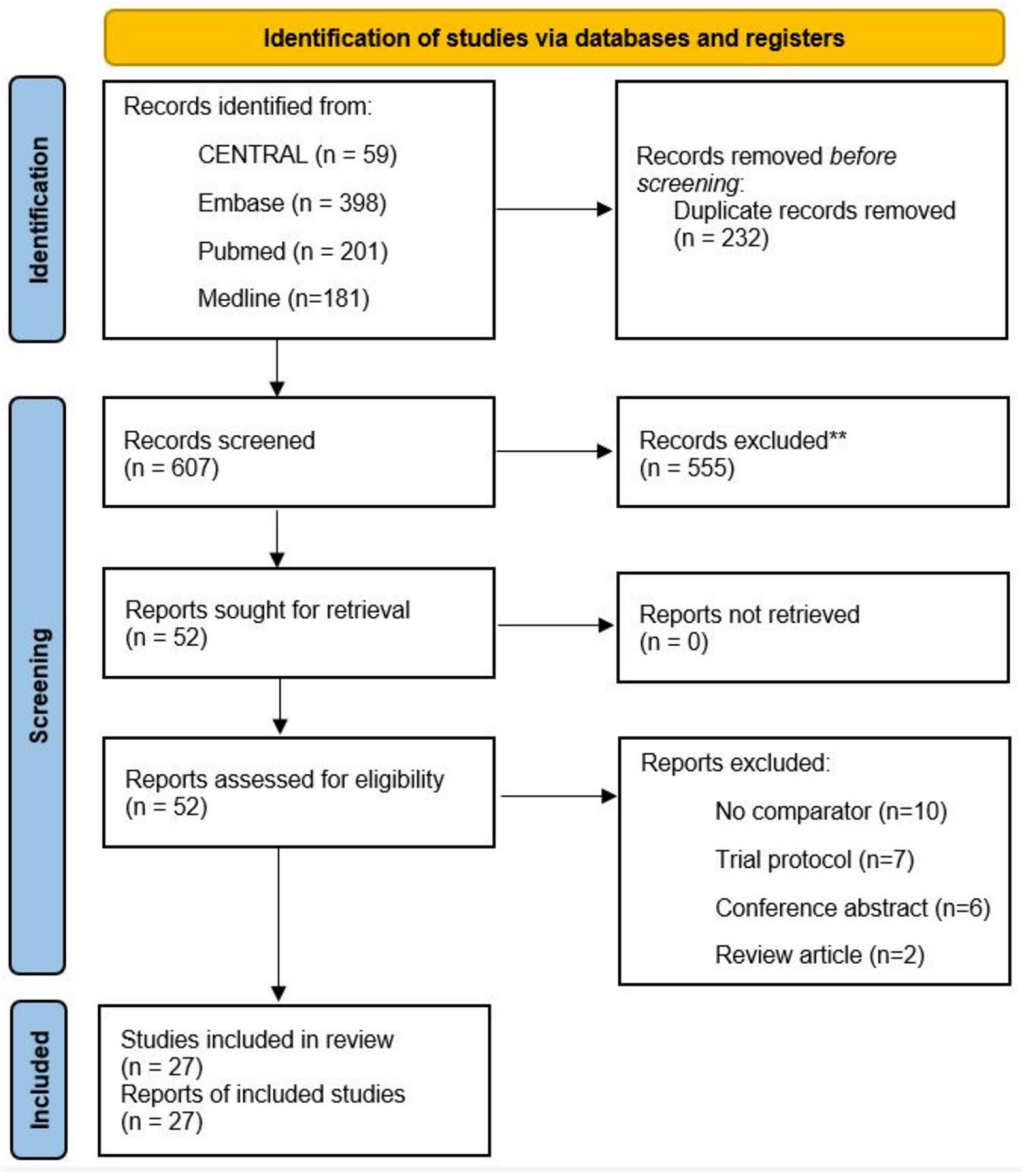
PRISMA flow diagram of study selection. Preferred Reporting Items for Systematic Reviews and Meta-Analyses (PRISMA) flow diagram outlining the study selection process. The number of records identified, screened, assessed for eligibility, and included in the final analysis are detailed at each stage

The search yielded 839 studies, of which 27 met inclusion criteria (Fig. 1). These comprised 15 retrospective observational studies, 4 prospective observational studies, and 8 RCTs. This encompassed 3752 patients (1763 MIS; 1989 BHC) of predominantly elderly cohorts (mean age 70.2 years MIS; 69.1 years BHC) and a male predominance across most studies (female proportion 31.0% MIS; 30.5% BHC) (Table 1). Minimally invasive evacuation most commonly involved TDC (56.2%), followed by SEPS (27.0%), with fewer studies employing YL-1 puncture needles (12.2%) and other percutaneous devices such as hollow screws (3.8%) or Integra Camino bolts (0.9%). Anticoagulant and/or antiplatelet exposure was common but variably reported. Baseline neurological severity was inconsistently reported using MGS, Glasgow Coma Scale (GCS), or modified Rankin Scale (mRS), precluding pooled comparison. Radiographic characteristics, including preoperative haematoma thickness, midline shift, CT density, and laterality were variably documented across studies (Table 2); where reported, mean preoperative haematoma thickness was comparable between groups (21.6 mm MIS; 21.1 mm BHC), with unilateral hypodense or mixed-density haematomas predominating. Mean follow-up was 17.8 months for MIS cohorts and also 17.8 months for BHC. No included study employed adjunctive MMAE (prespecified exclusion criterion), with all outcomes reflecting comparative performance of surgical drainage strategies alone. Methodological appraisal rated the overall review as high quality according to AMSTAR-2 criteria (Supplementary Table 2).

**Table 1:** Baseline characteristics of included studies comparing minimally invasive surgery (MIS) and burr hole craniostomy (BHC)

Author (year)	Country	Study design	Sample size (*n*)		Technique (access size)		Age (years) (mean ± SD or mean (range))		Sex (% female)		Comorbidities (n)		Anticoagulant/antiplatelet use (n)		SDH severity (MGS/GCS/mRS) (mean ± SD or n or mean (range))	
			MIS	BHC	MIS	BHC	MIS	BHC	MIS	BHC	MIS	BHC	MIS	BHC	MIS	BHC
Garber (2016)	USA	RC	15	87	TDC	Double	76.7	69.4	26.7	41.4	HTN (5); DM (3)	HTN (33); DM (18)	Antiplatelet (4); Anticoagulant (4)	Antiplatelet (24); Anticoagulant (11)	NR	NR
Williams (2001)	USA	RC	11	14	TDC	Single (15 mm)	63.0	57.4	NR	NR	NR	NR	NR	NR	MGS: 2	MGS: 2.3
Rughani (2010)	USA	RC	21	21	SEPS (6 mm)	Single or double	73.0	73.3	34	34	NR	NR	Total (6)	Total (4)	NR	NR
Smely (1997)	Germany	PC	33	33	TDC (5 mm)	Single (11 mm)	69.7 ± 12.7	70.0 ± 15.0	36.4	36.4	NR	NR	NR	NR	MGS 0: 0/33; MGS 1: 6/33; MGS 2: 19/33; MGS 3: 6/33; MGS 4: 2/33	MGS 0: 0/33; MGS 1: 6/33; MGS 2: 14/33; MGS 3: 10/33; MGS 4: 3/33
Muzii (2005)	Italy	RCT	22	24	TDC (5 mm)	Single or double (10 mm)	78.8 ± 8.2	76.3 ± 6.27	86.0	84.0	NR	NR	NR	NR	NR	NR
Lakshman (2022)	India	RCT	41	42	TDC	NR	NR	NR	78.0	65.0	Coagulation disorder (1); Abnormal liver function (1)	Coagulation disorder (3); Abnormal liver function (1)	NR	NR	NR	NR
Wan (2017)	China	RC	31	31	YL-1 (20 mm)	NR	72.5 (55–87)	73.6 (61–83)	38.7	41.9	CAD (5); CVD (6); HTN (10); DM (14); COPD (3); Seizures (1); CKD (4); Liver disease (2); Psychiatric (3); Alcoholism: (6)	CAD (8); CVD (7) HTN (22); DM (13); COPD: (4); Asthma (1); Tuberculosis (1); Seizure (2); CKD (1); Liver disease (2); Psychiatric (1); Parkinsonism (1); Anemia (2); Hypothyroidism (2); Alcoholism (7)	Warfarin (2)	Warfarin (1)	NR	NR
Thavara (2019)	India	RC	46	63	TDC (10 mm)	Single (25 mm)	73.4 ± 10.8	61.4 ± 13.2	30.4	23.8	NR	NR	Total (9)	Total (10)	GCS: 12.47 ± 2.95	GCS: 13.44 ± 2.23
Lin (2011)	China	RC	178	270	TDC	NR (12 mm)	63.2 ± 21.1	62.3 ± 24.5	13.5	19.3	HTN (11); DM (3)	HTN (6); DM (2)	NR	NR	NR	NR
Kim (2014)	Korea	RC	48	17	TDC (6 mm)	Single (15 mm)	67.9 (42–94)	70.6 (31–94)	41.7	52.9	NR	NR	Total (1)	Total (1)	MGS 0: 0/48; MGS 1: 37/48; MGS 2: 8/48; MGS 3: 3/48; MGS 4: 0/48	MGS 0: 0/17; MGS 1: 12/17; MGS 2: 2/17; MGS 3: 3/17; MGS 4: 0/17
Xu (2020)	China	RC	116	42	YL-1 (20 mm)	NR	65.4 ± 14.5	67.1 ± 13.8	14.7	16.7	HTN (23); DM (6)	HTN (31); DM (14)	Urokinase (45)	Urokinase (16)	NR	NR
Balser (2013)	USA	RC	29	44	SEPS	NR	76.6 ± 11.03	78.4 ± 8.4	0.0	0.0	NR	NR	Total (8)	Total (13)	NR	NR
Mohan (2022)	USA	RC	55	105	SEPS	NR	72.0 ± 11.1	71.0 ± 10.9	34.6	22.9	NR	NR	Total (24)	Total (46)	NR	NR
Goyal (2018)	India	RCT	20	20	TDC (4 mm)	Double (15 mm)	60.5	62.9	15.0	15.0	Alcoholism (15); HTN (11); DM (9)		Total (9)		NR	
Min (2017)	China	PC	67	6	TDC	NR	69.0 ± 18.0	61.0 ± 28.75	16.4	16.7	CVD (47); Malignancy (7); Alcoholism (3); Multiple (7)	CVD (48); Malignancy (7); Chronic alcohol (2); Multiple (4)	NR	NR	NR	NR
Eisenkolb (2025)	Germany	RCT	67	64	HS (4 mm)	Single (14 mm)	77.0 ± 10.6	77.0 ± 8.4	25.0	23.0	NR	NR	Antiplatelet (21); Anticoagulant (21); Both (1)	Antiplatelet (21); Anticoagulant (15); Both (2)	mRS: 1.0 (0–5)	mRS: 1.5 (0–5)
Wang (2016)	China	RC	68	53	YL-1 (3 mm)	NR (10 mm)	72.8 ± 11.2	71.2 ± 12.1	26.5	22.6	NR	NR	Total (0)	Total (0)	MGS 0: 0/68; MGS 1: 45/68; MGS 2: 17/68; MGS 3: 0/68; MGS 4: 0/68	MGS 0: 0/53; MGS 1: 32/53; MGS 2: 19/53; MGS 3: 2/53; MGS 4: 0/53
Certo (2019)	Italy	RC	15	30	Integra (5 mm)	Single	75.7	76.8	40.0	36.7	HTN (18); DM (6)	HTN (15); DM (6)	NR	NR	NR	NR
Wang (2017)	China	PC	38	45	TDC (2.5 mm)	Single (13 mm)	64.7 ± 11.2	67.9 ± 12.0	21.1	20.0	NR	NR	Total (6)	Total (6)	GCS 14–15: 34/38; GCS 9–13: 2/38; GCS < 8: 2/38	GCS 14–15: 34/45; GCS 9–13: 2/45; GCS < 8: 2/45
Gokmen (2008)	Turkey	RCT	38	32	TDC (4.5 mm)	Single (13 mm)	67.0 (35–98)		22.9		HTN (10)	HTN (5); DM (1)	NR	NR	NR	NR
Xu (2018)	China	RCT	20	20	TDC	NR (12 mm)	66.2 ± 10.1	66 ± 16.7	20.0	15.0	NR	NR	Aspirin (0)	Total (0)	mRS: 2.74 ± 1.49	mRS: 2.55 ± 1.47
Singh (2011)	India	RCT	48	52	TDC (4 mm)	Double (15 mm)	59.8	61.2	10.4	9.6	CVD or DM (51)	CVD or DM (49)	Anticoagulant (1)		MGS: 1.95 GCS: 11.87	
Duerinck (2022)	Belgium	RCT	82	79	TDC	NR	74.3 ± 14.8	74.3 ± 13.0	37.8	39.2	NR	NR	Total (44)	Total (41)	MGS 0: 6/82; MGS 1: 38/82; MGS 2: 33/82; MGS 3: 4/82; MGS 4: 1/82	MGS 0: 6/79; MGS 1: 41/79; MGS 2: 22/79; MGS 3: 9/79; MGS 4: 1/79
Gabarros (2000)	Spain	RC	105	83	TDC	NR	66.5 (17–86)		29.0		NR	NR	Total (1)		NR	NR
Peters (2023)	USA	RC	110	35	TDC	NR	77.5 ± 7.1	78.8 ± 7.8	25.5	51.4	HTN (196); DM (124); AF (83); CAD (84); CHF (52); TCP (19)	HTN (360); DM (184); AF (139); CAD (133); CHF (90); TCP (19)	Antiplatelet (48); Anticoagulant (14)	Antiplatelet (15); Anticoagulant (4)	GCS 13–15: 97/110; GCS 9–12: 9/110; GCS 3–8: 4/110	GCS 13–15: 33/35; GCS 9–12: 1/35; GCS 3–8: 1/35
Flint (2017)	USA	RC	371	659	SEPS	Single	75.0 (65–82)	76.0 (67–83)	30.7	29.6	NR	NR	NR	NR	NR	NR
Lee (2016)	Korea	PC	68	18	TDC	NR	68.2 ± 13.9	67.8 ± 16.7	51.2	25.0	HTN (5); DM (3)	HTN (33); DM (18)	NR	NR	MGS 1: 21/68; MGS 2: 36/68; MGS 3: 8/68; MGS 4: 2/68	MGS 1: 6/18; MGS 2: 11/18; MGS 3: 1/68; MGS 4: 0/68

*RC* Retrospective Cohort, *PC* Prospective Cohort, *RCT* Randomised Control Trial, *SDH* Subdural Haematoma, *TDC* Twist Drill Craniostomy, *SEPS* Subdural Evacuating Port System, *YL-1* YL-1 Puncture Needle, *HS* Hollow Screw, *Integra* Integra Percutaneous Drainage System, *HTN* Hypertension, *DM* Diabetes Mellitus, *CVD* Cardiovascular Disease, *CAD* Coronary Artery Disease, *CKD* Chronic Kidney Disease, *COPD* Chronic Obstructive Pulmonary Disease, *CHF* Congestive Heart Failure, *TCP* Thrombocytopaenia, *N* Not Reported

**Table 2:** Radiographic and treatment characteristics of included studies comparing minimally invasive surgery (MIS) and burr hole craniostomy (BHC)

Author (year)	Preoperative haematoma maximum thickness (mm) (mean ± SD or mean (range))		Midline shift (mm or number of patients reported with midline shift) (mean ± SD or mean (range))		CT density (n)		Laterality of cSDH (unilateral/bilateral)		Anaesthesia (n)		Drain type and drainage duration (days)		Irrigation		Complications breakdown (n)		Follow-up (months)	
	MIS	BHC	MIS	BHC	MIS	BHC	MIS	BHC	MIS	BHC	MIS	BHC	MIS	BHC	MIS	BHC	MIS	BHC
Garber (2016)	17.5	17	13/15	80/87	NR	NR	15/0	75/12	LA (15)	GA (87	Subdural	Subdural	NR	NR	Acute SDH (1); recurrent SDH (1)	Recurrent SDH (6)	34.0	
Williams (2001)	NR	NR	NR	NR	NR	NR	NR	NR	LA (11)	LA (14)	Subdural (1–2)	Subdural (1–2)	No	Saline	NR	NR	1.7	1.2
Rughani (2010)	NR	NR	NR	NR	NR	NR	15/6	15/6	LA (18); GA (3)	LA (4); GA (17)	Subdural (2)	Subdural drain (1.8)	No	Saline	Seizure (1); acute bleed (1)	Seizure (1)	2.0	1.5
Smely (1997)	21.0	22.0	11/33	9/33	Hypo- (23); Iso- (13)	Hypo-(26); Iso- (14)	30/3	26/7	LA (33)	LA (33)	Subdural (3.1)	Robinson with closed system (5.7)	Saline	Saline	0	Infection (6)	81.0	82.0
Muzii (2005)	NR	NR	NR	NR	Hypo- (9); Iso- (5); Mixed (8)	Hypo- (13); Iso- (6); Mixed (5)	18/4	20/4	LA (22)	LA (24)	Subdural (2.6)	Subdural (1.6)	No	Saline	NR	NR	2.0	
Lakshman (2022)	NR	NR	NR	NR	NR	NR	33/9	37/4	NR	NR	NR	NR	NR	NR	NR	NR	0.5	
Wan (2017)	21.0	22.0	NR	NR	Hyper- (9); Hypo- (17); Mixed (5)	Hyper- (7); Hypo- (13); Mixed (11)	31/0	31/0	LA (31)	LA (31)	Catheter (3–5)	Subdural (3–5)	Saline	Saline	Pneumocephalus (1)	Pneumocephalus (7)	3.0	
Thavara (2019)	22.2 ± 4.5	17.2 ± 4.3	45/46	59/63	NR	NR	46/0	63/0	LA (46)	GA (63)	Subdural (1)	Subdural (1)	No	Saline	Acute bleed (4)	Seizures (1); pneumocephalus (1); acute bleed (3)	NR	NR
Lin (2011)	NR	NR	108/178	194/270	Hypo- (14); Iso- (12); Hyper- (44); Mixed (108)	Hypo- (0); Iso- (14); Hyper- (116); Mixed (140)	NR	NR	LA (178)	LA (222/270); GA (48/270)	Closed loop (3)	Subdural (3)	No	Physiologic saline	Recurrent SDH (14)	Infection (4); tension pneumocephalus (6); brain injury (5); epilepsy (9); recurrent SDH (32)	NR	NR
Kim (2014)	22.5 (10.8–39.9)	17.3 (8.9–23.1)	11 (0–22.48)	8 (2.12–16.08)	Hypo- (12); Iso- (18); Hyper- (11); Mixed (7)	Hypo- (4); Iso- (8); Hyper- (1); Mixed (4)	37/11	13/4	LA (48)	LA (17)	Subdural (3.8)	Subdural (3.4)	No	NR	Pneumocephalus (3); misplaced catheter (1)	Pneumocephalus (5)	NR	NR
Xu (2020)	NR	NR	NR	NR	NR	NR	98/18	36/6	LA (116)	GA (42)	Subdural (2–4)	Subdural	Saline	NR	NR	NR	3.0	
Balser (2013)	NR	NR	NR	NR	NR	NR	23/6	38/6	LA (29)	GA (16); LA (28)	NR	NR	NR	NR	NR	NR	36.0	
Mohan (2022)	18.4 ± 6.3	18.6 ± 7.8	NR	NR	NR	NR	55/0	105/0	LA (55)	GA (105)	SEPS	Subdural or subgaleal (1–2)	NR	NR	Seizure (4)	Seizure (7)	96.0	
Goyal (2018)	20.85	23	8.25	9.15	NR	NR	20/0	20/0	LA (20)	LA (20)	Soft ventricular catheter	Subdural	Saline	Saline	Tension pneumocephalus (6); postoperative fever (3); infection (2); seizure (1)		6.0	
Min (2017)	20.6 ± 6.9	17.7 ± 2.2	NR	NR	NR	NR	0/67	0/6	LA (67)	LA (6)	Closed system	Closed system	Saline	NR	NR	NR	3.0	
Eisenkolb (2025)	NR	NR	NR	NR	NR	NR	NR	NR	LA (67)	GA (64)	Closed-loop (1–4)	Subdural (1–4)	Ringer’s	Ringer’s	Acute SDH (2)	Acute SDH (1); temporary hemiparesis (1)	1.5	1.5
Wang (2016)	21.1 ± 5.1	21.8 ± 4.9	11.02 ± 4.13	11.23 ± 4.08	Hypo- (9); Iso- (46); Mixed (13)	Hypo- (15); Iso- (31) Mixed (7)	56/12	45/8	LA (67); GA (1)	LA (11); GA (42)	Subdural (1–3)	Subdural (1–3)	Water	Saline	Drill laceration (1); pneumocephalus (9)	Acute EDH (2); acute SDH (1); pneumonia (3); pneumocephalus (46)	1.0	
Certo (2019)	33	38.5	NR	NR	NR	NR	NR	NR	LA (15)	LA (15); GA (15)	Subdural (2–3)	Subdural (2–3)	Isotonic	Isotonic	0	NR	14.8	
Wang (2017)	NR	NR	< 5: 14 (36.8%) 5—10: 16 (42.1%) > 10: 8 (21.1%)	< 5: 13 (28.9%) 5–10: 17 (37.8%) > 10: 15 (33.3%)	NR	NR	28/0	38/0	LA (68)	LA (53)	Subdural (3.1 ± 1.0)	Subdural (2.5 ± 0.9)	No	Warm saline	Infection (1); pneumonia (1)	Infection (1); epilepsy (1); pneumonia (1)	3.0	
Gokmen (2008)	18		10		Hypo- (12); Iso- (18); Mixed (17)		38/0	32/0	LA or GA	LA or GA	Subdural (2)	Subdural (2)	No	Saline	Drill fail (1); drain kinking (1); CN6 paresis (1)	Contralateral cSDH (1)	6.0	
Xu (2018)	NR	NR	NR	NR	NR	NR	NR	NR	NR	NR	Subdural (2)	Subdural (2)	No	Warm saline	Infection (1)	Infection (1)	3.0	
Singh (2011)	22.1	19.8	10.5	9.86	NR	NR	48/0	52/0	LA (48)	GA (52)	Closed system subdural	Closed system subdural	Gentamicin mixed saline	Gentamicin mixed saline	Infection (1); meningitis (1); haematoma (5)	Infection: (3); haematoma (4)	3.0	
Duerinck (2022)	22.4 ± 8.0	20.8 ± 5.7	7.5 ± 4.7	6.7 ± 4.6	Hypo- (29); Iso- (20); Mixed (33)	Hypo- (33); Iso- (13); Mixed (33)	60/22	60/19	GA (82)	GA (79)	Subdural drain (2)	Subdural drain (2)	NR	NR	Epilepsy (7); pneumonia (4); stroke (1) IPH (1); misplaced drain (5); hydrocephalus (1); empyema (1)	Epilepsy (6) pneumonia (5); heart failure (1); renal failure (2); acute SDH (2); IPH (1); misplaced drain (1); cerebral edema (1); EDH (1)	6.0	
Gabarros (2000)	NR	NR	NR	NR	NR	NR	78/5		LA or GA	LA or GA	NR	NR	NR	NR	NR	NR	NR	
Peters (2023)	NR	NR	NR	NR	NR	NR	100/10	32/3	LA (110)	LA (35)	NR	NR	NR	NR	UTI (14); pneumonia (8); DVT (2); PE (2); acute bleed (4)	UTI (5) pneumonia (3); DVT (2); PE (1); acute bleed (1)	13.0	
Flint (2017)	NR	NR	NR	NR	NR	NR	336/35	536/123	LA (371)	LA (501); GA (168)	SEPS	Jackson-Pratt or subdural	NR	NR	Seizures (6); acute bleed (7)	Seizures (15); pneumocephalus (5); acute bleed (14)	72.0	
Lee (2016)	NR	NR	NR	NR	NR	NR	58/10	18/0	LA (68)	NR	Subdural (3.2 ± 1.0)	Subdural (3.4 ± 0.8)	No	No	0	0	> 3	

*LA* Local Anaesthesia, *GA* General Anaesthesia, *SDH* Subdural Haematoma, *EDH* Extradural Haematoma, *IPH* Intraparenchymal Haematoma, *UTI* Urinary Tract Infection, *DVT* Deep Vein Thrombosis, *PE* Pulmonary Embolism, *NR* Not Reported

### Primary outcomes

**Fig. 2:**
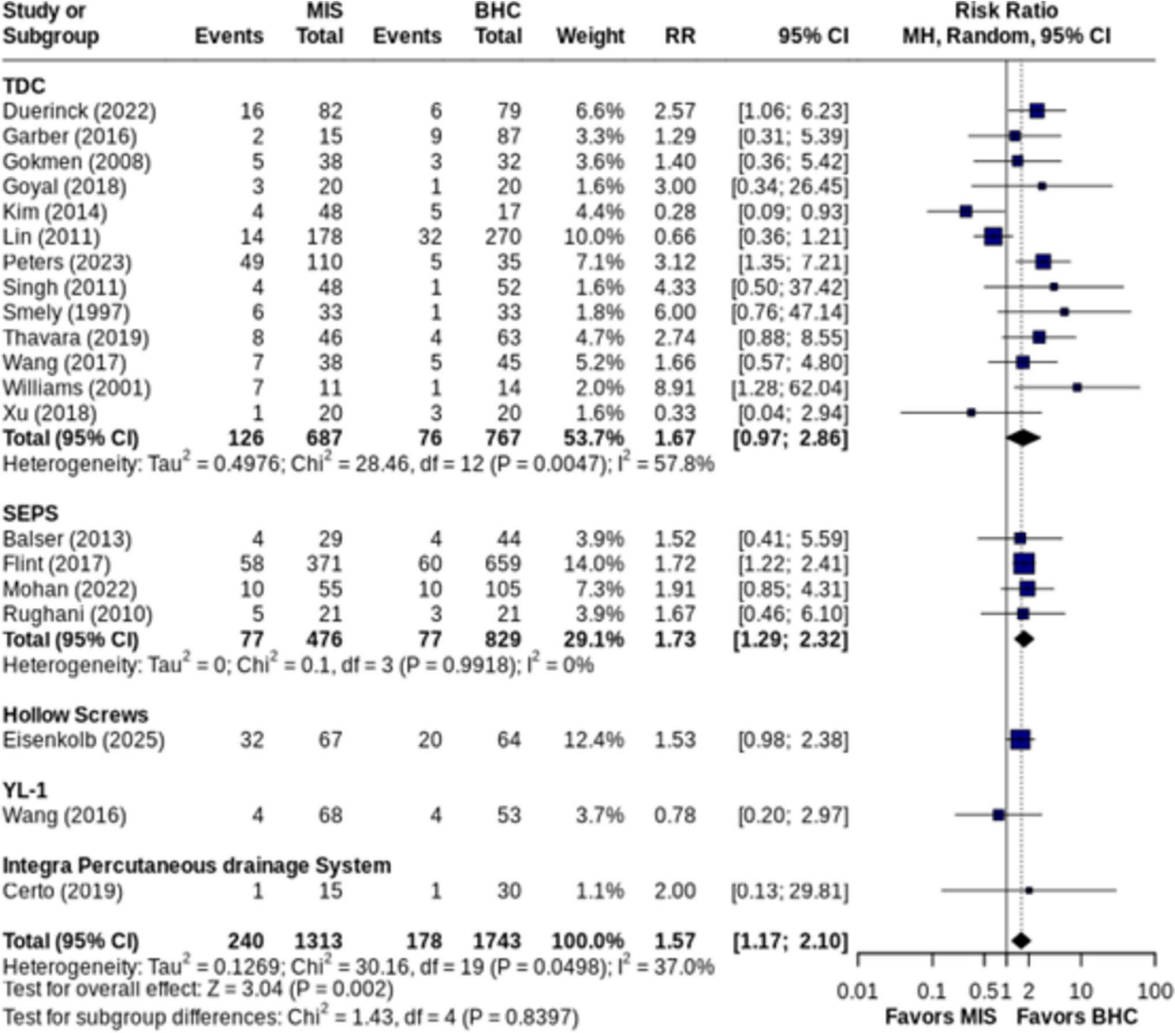
Forest plot comparing reoperation in patients following minimally invasive surgery (MIS) or burr hole craniostomy (BHC) with subgroup analysis by device type. Abbreviations: TDC: Twist Drill Craniostomy; SEPS: Subdural Evacuating Port System; YL-1: YL-1 Puncture Needle

Twenty studies [1, 2, 4, 6, 8, 11–13, 16, 24, 29, 34, 39, 44, 45, 50, 52, 53, 56, 57] (74.1%) reported reoperation, with prespecified device-level subgroup analyses (SEPS, TDC, and YL-1) performed against BHC. Reoperation occurred in 18.3% of MIS patients (240/1313) and 10.2% of BHC patients (178/1743), with a pooled RR of 1.57 (95% CI: 1.17–2.10; *P* < 0.01; I^2^ = 37.0%). Leave-one out sensitivity analysis confirmed the stability of this finding. Device-specific subgroup analysis showed an increased reoperation risk with SEPS when analysed separately against BHC (RR 1.73, 95% CI: 1.29–2.32; I^2^ = 0.0%) (Fig. 2). RCT-specific subgroup analysis demonstrated directional consistency with the main effect (RR 1.67, 95% CI: 1.07–2.59; I^2^ = 0.0%).

**Fig. 3:**
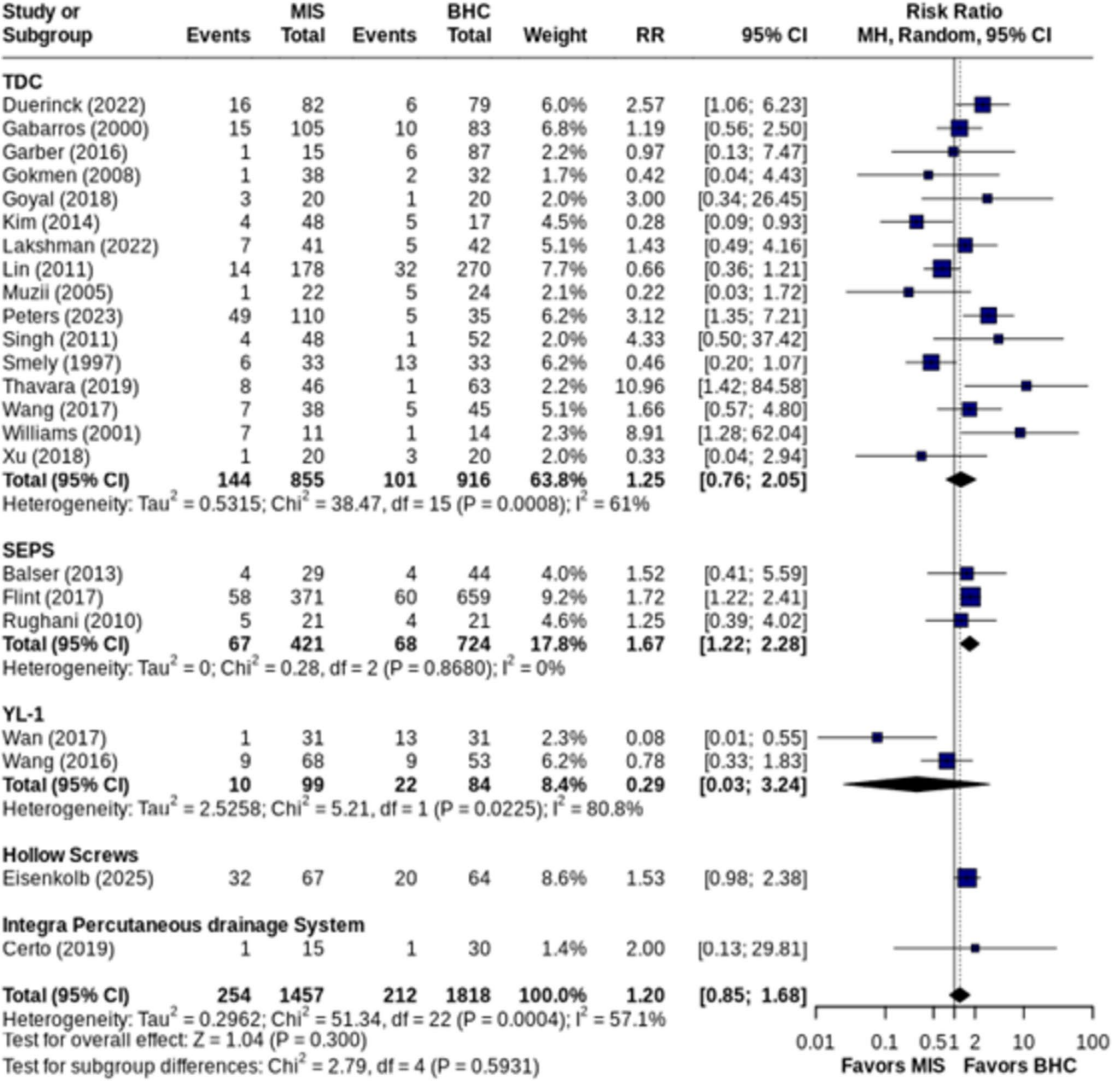
Forest plot comparing recurrence in patients following minimally invasive surgery (MIS) or burr hole craniostomy (BHC) with subgroup analysis by device type. Abbreviations: TDC: Twist Drill Craniostomy; SEPS: Subdural Evacuating Port System; YL-1: YL-1 Puncture Needle

Twenty-three studies [1, 2, 4, 6, 8, 9, 11–13, 16, 19, 24, 30, 34, 39, 44, 45, 50–53, 56, 57] (85.2%) reported recurrence. Recurrence occurred in 17.4% of MIS patients (254/1457) and 11.2% of BHC patients (212/1818), with pooled RR of 1.20 (95% CI: 0.85–1.68; *P* = 0.30; I^2^ = 57.1%). Device-specific subgroup analysis showed higher recurrence with SEPS relative to BHC (RR 1.67, 95% CI: 1.22–2.28; I2 = 0.0%) (Fig. 3). RCT-specific subgroup analysis demonstrated directional consistency with the main effect (RR 1.52, 95% CI: 0.95–2.45; I^2^ = 22.5%).

**Fig. 4:**
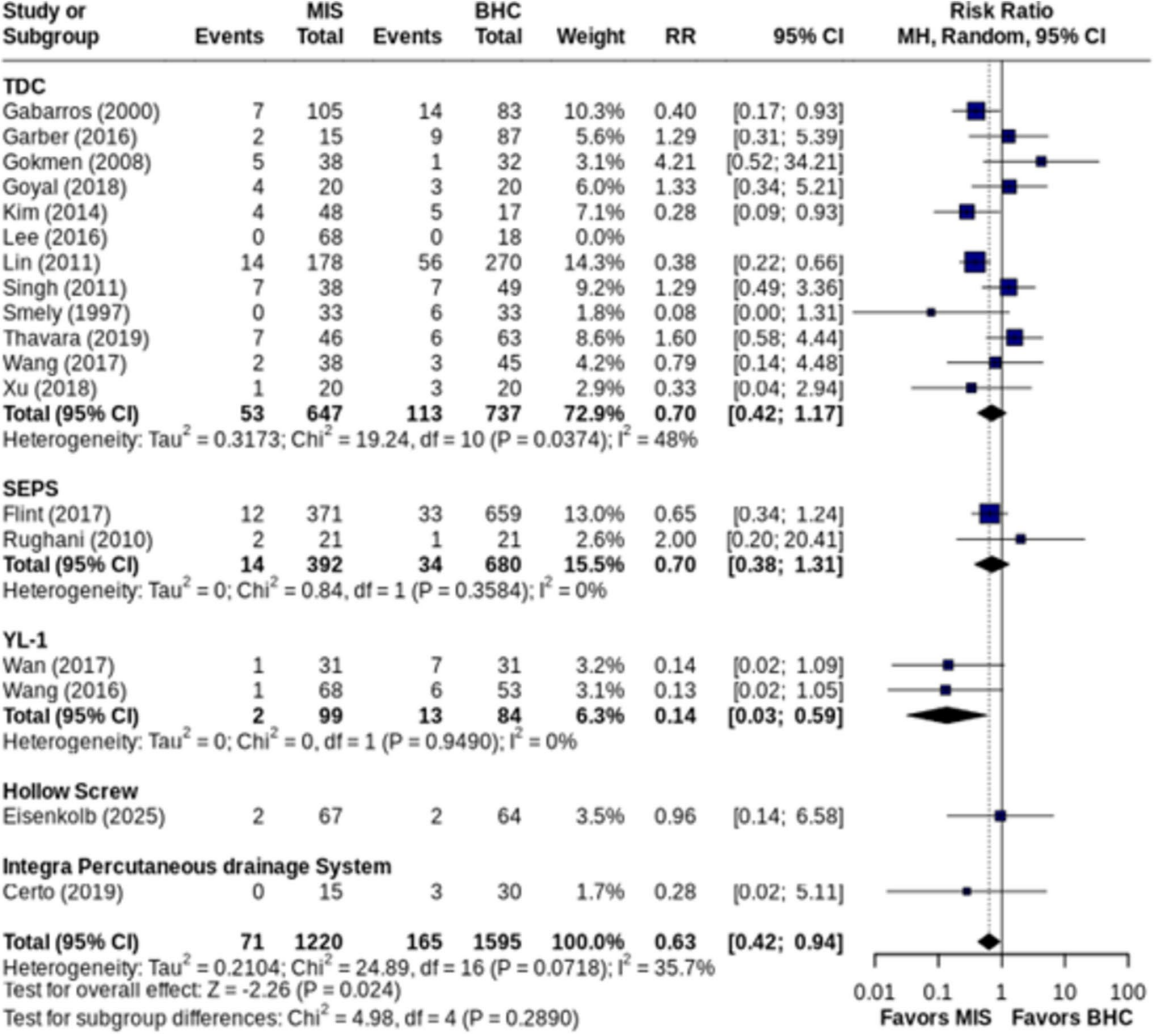
Forest plot comparing overall complications in patients following minimally invasive surgery (MIS) or burr hole craniostomy (BHC) with subgroup analysis by device type. Abbreviations: TDC: Twist Drill Craniostomy; SEPS: Subdural Evacuating Port System; YL-1: YL-1 Puncture Needle

Eighteen studies [2, 6, 8, 9, 11–13, 16, 20, 24, 39, 44, 45, 50–53, 57] (66.7%) reported overall complications (Table 2). Overall complications occurred in 5.8% of MIS patients (71/1220) and 10.3% of BHC patients (165/1595), with a pooled RR was 0.63 (95% CI: 0.42–0.94; *P* < 0.05; I^2^ = 35.7%). Leave-one out sensitivity confirmed the stability of this finding. Device-specific subgroup analysis showed lower complication risk with YL-1 compared to BHC (RR 0.14; 95% CI: 0.03–0.59; I^2^ = 0.0%) (Fig. 4). RCT-specific subgroup analysis demonstrated directional consistency with the main effect (RR 1.25, 95% CI: 0.57–2.71; I^2^ = 0.0%).

### Secondary outcomes

Nineteen studies [4, 8, 9, 12, 13, 19, 20, 24, 30, 34, 39, 44, 45, 50–53, 56, 57] (70.4%) reported mortality. Mortality occurred in 7.7% of MIS patients (104/1351) and 7.4% of BHC patients (117/1587), with a pooled RR of 1.21 (95% CI: 0.93–1.57; *P* = 0.15; I^2^ = 0.0%). Device-specific subgroup analysis demonstrated no significant differences (*P* = 0.92) (Supplementary Fig. 1).

Ten studies [2, 9, 12, 13, 24, 28, 44, 52, 56, 57] (37.0%) reported complete clinical resolution. This occurred in 84.4% of MIS patients (480/569) and 79.6% of BHC patients (461/579), with a pooled RR of 0.98 (95% CI: 0.88–1.10; *P* = 0.75; I^2^ = 80.9%). Device-specific subgroup analysis demonstrated no significant differences (*P* = 0.98) (Supplementary Fig. 2).

### Complications

Eleven studies [4, 6, 8, 11, 12, 34, 39, 44, 50–52] (40.7%) reported postoperative intracranial bleeding. Postoperative intracranial bleeding occurred in 3.0% of MIS patients (27/897) and 2.9% of BHC patients (35/1173), with a pooled RR of 1.04 (95% CI: 0.55–1.98; *P* = 0.90; I^2^ = 21.2%). Device-specific subgroup analysis demonstrated no significant differences (*P* = 0.46) (Supplementary Fig. 3).

Six studies [8, 16, 24, 50–52] (22.2%) reported pneumocephalus. Pneumocephalus occurred in 17.5% of MIS patients (13/742) and 6.4% of BHC patients (70/1093), with a pooled RR of 0.16 (95% CI: 0.10–0.72; *P* < 0.001; I^2^ = 0.0%) (Supplementary Fig. 4). Leave-one out sensitivity analysis confirmed stability of this finding.

Seven studies [4, 8, 24, 44, 45, 53, 57] (25.9%) reported wound infection. Wound infection occurred in 0.1% of MIS patients (4/770) and 0.1% of BHC patients (16/1155), with a pooled RR of 0.42 (95% CI: 0.14–1.25; *P* = 0.12; I^2^ = 0.0%) (Supplementary Fig. 5).

Seven studies [4, 8, 24, 29, 39, 50, 52] (25.9%) reported seizure. Seizure occurred in 2.1% of MIS patients (17/818) and 3.2% of BHC patients (40/1253), with a pooled RR of 0.77 (95% CI: 0.44–1.35; *P* = 0.36; I^2^ = 0.0%) (Supplementary Fig. 6).

### Functional outcomes

Six studies [4, 13, 16, 20, 45, 52] (22.2%) reported MGS. Mean preoperative MGS was 1.75 for MIS patients (319 patients) and 1.83 for BHC patients (220 patients), with an MD of 0.06 (95% CI: −0.24–0.13; *P* = 0.56; I^2^ = 46.1%) (Supplementary Fig. 7). Mean postoperative MGS was 0.40 for MIS patients (238 patients) and 0.40 for BHC patients, with an MD of −0.00 (95% CI: −0.16–0.16; *P* = 0.99; I^2^ = 41.7%) (Supplementary Fig. 8). However, functional outcomes were variably defined and inconsistently reported with respect to follow-up timing across studies, limiting comparability and sensitivity to detect longer-term differences.

### Technical outcomes

Eight studies [4, 6, 16, 28, 50, 52, 53, 58] (29.6%) reported OT. Mean OT was 24.5 min for MIS patients (532 patients) and 51.4 min for BHC patients (369 patients), with an MD of −24.5 (95% CI: −31.38–−17.55; *P* < 0.001; I^2^ = 96.0%) (Supplementary Fig. 9). Leave-one out sensitivity analysis confirmed stability of these findings.

LOS was reported by twelve studies [1, 6, 8, 16, 28, 29, 34, 50–52, 57, 58] (44.4%). Mean LOS was 8.5 days for MIS (1028 patients) and 10.6 days for BHC, with an MD of −1.97 (95% CI: −3.36–−0.58; *P* < 0.01; I^2^ = 93.3%) (Supplementary Fig. 10). Leave-one out sensitivity analysis confirmed the stability of these findings.

### Publication bias, risk of bias, and quality assessment

Funnel plots showed symmetry with all studies found beneath the 95% CI pyramidal lines (Supplementary Fig. 11) and Egger’s regression test demonstrated no evidence of small-study effects (*P* = 0.80). Among observational studies, 3 were low risk of bias, 9 moderate risk, and 7 serious risk, with confounding the most common concern (Supplementary Table 3). All RCTs were judged to have “some concerns” on RoB 2 (Supplementary Table 4). Certainty of evidence was graded as moderate for the primary outcomes, with downgrading due to risk of bias (Supplementary Table 5). Quality assessment was conducted on five previous systematic reviews, four of which were rated as critically low in quality by AMSTAR-2 (Supplementary Table 6).

## Discussion

MIS has become an increasingly attractive alternative to BHC for cSDH, driven by an understandable desire to reduce operative trauma, avoid general anaesthesia, and streamline perioperative care. Yet biological plausibility does not guarantee biological equivalence. Across 27 studies and 3752 patients, this meta-analysis demonstrates a consistent pattern: MIS offers procedural efficiency and reduces perioperative morbidity, at the expense of increased reoperation and recurrence risk (particularly for SEPS), reflecting fundamental mechanistic constraints intrinsic to percutaneous drainage platforms. BHC, though more invasive, remains the more durable intervention. The neurosurgical community is therefore confronted with a nuanced trade-off between immediacy and completeness of evacuation, between procedural minimalism and pathophysiological adequacy. These findings are directionally consistent with previous literature [26, 35, 59].

### Mechanistic drivers of higher reoperation and recurrence after MIS

The central question is why MIS underperforms in durability despite achieving immediate decompression. The answer may lie in the underlying biology of cSDH. The chronic subdural cavity is not a passive space, but a dynamic, inflammatory system characterised by fragile neovasculature, high fibrinolytic activity, angiogenic signalling, and exudative protein flux [15]. Residual membrane surfaces continue to generate protein-rich fluid through cyclical bleeding and osmotic gradients even after apparent decompression [5, 54].

MIS platforms, whether twist-drill, SEPS, or YL-1, are fundamentally constrained by their geometry and offer limited ability to disrupt septations, irrigate residual haematoma, or meaningfully access organised clot. Their drainage is predominantly passive, relying on small-calibre fenestrations that are prone to obstruction by clot fragments or membrane debris. This sets the stage for incomplete clearance of the biochemical milieu that sustains reaccumulation [42]. Moreover, MIS-associated reaccumulation is mechanistically linked to air entrainment dynamics. BHC allows controlled irrigation that reduces postoperative pneumocephalus, whereas twist-drill and SEPS techniques create smaller conduits more susceptible to negative-pressure air ingress [8]. Postoperative pneumocephalus may transiently prevent brain re-expansion, preserving the subdural dead space that permits recurrent fluid accumulation [21]. This phenomenon is consistent with recurrence risk observed in SEPS cohorts [18].

In addition, many MIS protocols are deployed under local anaesthesia at the bedside, where constraints on patient positioning, irrigation volume, and intraoperative imaging may further limit completeness of evacuation [8, 11, 50]. These practical restrictions compound the intrinsic mechanical limitations of percutaneous systems and likely contribute to residual haematoma volume, suboptimal membrane disruption, and early reaccumulation – factors that mechanistically align with higher reoperation risk observed in our analysis.

The device-specific signal of SEPS performing worst likely reflects a combination of small-bore fenestrations, minimal penetration depth, and reduced capacity to handle loculated collections [4]. Conversely, the YL-1 system’s comparatively lower complication risk may reflect differences in catheter stiffness, geometry, and tissue interface, although heterogeneity across studies limits definitive inference [49].

### Why burr hole craniostomy remains more durable

BHC achieves superior long-term control in this meta-analysis, and we hypothesize it does so by addressing both the mechanical and biological drivers of cSDH. The larger dural opening permits more effective irrigation, limited disruption of fragile septations, and improved evacuation of organised clot, facilitating reductive of the biologically active subdural environment. By reducing residual haematoma volume and membrane contact surface, BHC attenuates the biological drive for continued exudation and microhaemorrhage. Additionally, BHC facilitates immediate brain re-expansion by evacuating air and lower subdural surface tension. Brain re-expansion is not merely radiological – its restoration collapses the inflammatory cavity, re-establishes normal CSF-parenchymal pressure gradients, and mechanistically opposes recurrence. MIS, by contrast, often achieves a large proportion of its evacuation at the time of device placement, with subsequent drainage occurring passively over the following 24–72 h, leaving a prolonged window during which membrane-driven reaccumulation may outpace further clearance. These mechanistic advantages explain why BHC consistently demonstrates lower reoperation risk across the literature [27] and in this meta-analysis. An important future study to corroborate this hypothesis would definitively investigate the impact of membranolysis during BHC for cSDH. Indeed, a prior meta-analysis [40] suggests there may be benefit of membranolysis with regard to cSDH recurrence rates, and a prospective study evaluating this would be of utmost importance.

### Perioperative advantage of MIS: reduced complications, shorter procedures, and early mobilisation

Despite inferior durability, MIS offers tangible benefits, particularly for frail older adults with multimorbidity. MIS techniques avoid general anaesthesia, reduce soft-tissue dissection, and minimise dural manipulation. This translates into lower wound morbidity and fewer systemic complications, consistent with significant reduction in overall complications observed in our pooled analysis. Procedural times are dramatically shorter, often by 20–30 min, reflecting the simplicity of percutaneous access. This efficiency not only reduces anaesthetic exposure but also enhances throughput in capacity-constrained centres [14]. However, these technical outcomes exhibited substantial heterogeneity (I^2^ > 90%), indicating that pooled MDs for OT and LOS should be interpreted as context-dependent averages rather than universally generalisable effects, and are likely influenced by centre-specific workflows, anaesthetic practices, and discharge protocols.

### Device-specific performance: SEPS, twist-drill, and YL-1

Device heterogeneity as a central explanatory theme. As demonstrated by device-specific subgroup analyses comparing SEPS, TDC, and YL-1 systems individually against BHC, SEPS is consistently associated with higher reoperation and recurrence, likely reflecting intrinsic mechanical limitations such as shallow penetration depth, reliance on passive drainage, and vulnerability to clot obstruction. TDC systems perform modestly better but still lack the versatility of BHC. The YL-1 device, originating primarily from East Asian cohorts, shows favourable complication profiles, but these results should interpreted cautiously due to limited generalisability and heterogeneous perioperative protocols. This mechanistic advantage may stem from its stiffer lumen, larger drainage calibre, and design that mitigates kinking. These findings support a shift from viewing MIS as a monolithic category to a more granular, device-specific appraisal. However, these device-level signals should be interpreted cautiously, as several subgroup estimates are derived from relatively small numbers of studies and unadjusted comparisons, and are therefore hypothesis-generating rather than definitive. Further stratification beyond this level was not considered methodologically robust given the limited number of studies within each subgroup.

### Equivalent mortality and functional outcomes: understanding the physiological ceiling

Despite durability differences, mortality and functional outcomes were similar between MIS and BHC. Several explanations are biologically plausible. First, most patients with cSDH experience substantial neurological improvement once mass effect is relieved (regardless of technique), meaning the initial decompression phase dominates the recovery curve. Second, mortality in cSDH is often driven by comorbidities rather than the haematoma itself. Therefore, the marginal differences in surgical strategy may not materially influence outcome. This convergence suggests that recurrence, though an important quality-of-care metric, rarely translates into mortality, particularly when managed promptly. Instead, its primary impacts are on resource utilisation, quality of life, and cognitive recovery trajectories. Importantly, the interpretation of functional equivalence is constrained by heterogeneity in outcome definition and timing. Many included studies assessed functional status at discharge or early follow-up, potentially underestimating the cumulative impact of recurrence and reoperation of longer-term recovery trajectories. Standard scales such as MGS, while clinically pragmatic, may lack granularity to detect subtle but meaningful differences in cognitive recovery, independence, or quality of life.

These findings raise an important question: are we over-emphasising recurrence relative to patient-centred outcomes? We would argue that recurrence should not be interpreted solely as a surrogate for functional failure, but rather as a composite marker of treatment durability, patient burden, and healthcare utilisation. Even when functional outcomes ultimately converge, recurrence introduces additional interventions, delays rehabilitation, and increases cumulative risk exposure, particularly in frail populations.

### Health-system and economic implications

A critical question is not only what works, but what works for whom and at what cost. MIS offers reduced OT and LOS – a significant advantage for overstretched health systems [14]. However, while direct device-level cost comparisons (particularly for bedside procedures) remain poorly characterised in the literature, these procedural gains must still be weighed against downstream resource utilisation associated with reoperations, repeat imaging, and extended follow-up. For high-volume centres, a hybrid paradigm may emerge: MIS as a first-line intervention for frail or high-risk patients, and BHC for those with septated, organised, or high volume collections, or when preventing recurrence is paramount. Such stratified approaches reconcile physiological, logistical, and economic priorities. For example, a health system with a limited number of acute operating rooms may benefit from a system incorporating first-line bedside MIS evacuation in order to relieve pressure promptly. Future economic evaluations should incorporate both index procedure efficiency and recurrence-driven utilisation, rather than assuming that shorter procedures or bedside delivery translate directly into lower overall cost. From a health-economic perspective, these trade-offs are highly relevant. While MIS reduces OT and LOS, its higher reoperation risk may offset these gains through repeat admissions and interventions. In this context, number needed to treat frameworks and cost-effectiveness analyses become essential to determine whether upfront procedural efficiency translates into net system benefit.

### Integration with evolving adjuncts including middle meningeal artery embolisation

The emergence of MMAE reframes aspects of cSDH management but does not eliminate the strategic differences between MIS and BHC. Accordingly, studies incorporating adjunctive MMAE were prespecified as an exclusion criterion in the present analysis, ensuring that reported outcomes reflect the comparative performance of surgical evacuation strategies alone. MMAE addresses the biological component (reducing membrane vascularity and exudation) but does not compensate for inadequate mechanical evacuation. MIS combined with MMAE has been proposed as a potential hybrid strategy, pairing rapid decompression with biological stabilisation. However, whether embolisation can meaningfully compensate for incomplete mechanical evacuation remains unproven, and this paradigm therefore remains speculative pending rigorous comparative trials. This is particularly relevant in the context of functional outcomes, as MMAE may preferentially reduce recurrence risk without directly influencing early neurological recovery. As such, future treatment paradigms may decouple intermediate decompressive success from long-term durability, reinforcing the need to interpret functional outcomes, recurrence, and resource utilisation as interdependent but distinct domains.

### Methodological considerations

This review was conducted with high methodological rigour, including adherence to PRISMA and AMSTAR-2 standards, prospective protocol registration, and pre-specified sensitivity and subgroup analyses. Consistency across leave-one-out sensitivity analyses and low to moderate heterogeneity for most outcomes supports the robustness of the principal findings.

Nonetheless, several limitations warrant cautious interpretation. Although prespecified subgroup analyses by MIS device type and restriction to RCTs were undertaken to mitigate device heterogeneity and confounding by indication, residual selection bias cannot be fully excluded. The evidence base is largely observational and retrospective, introducing unavoidable risks of selection and treatment allocation bias that cannot be fully eliminated despite analytic safeguards [33]. Substantial heterogeneity in operative technique, drainage protocols, and follow-up practices limits cross-study comparability. In particular, technical outcomes such as OT and LOS demonstrated marked inter-study heterogeneity, reinforcing that these efficiency gains are sensitive to local practice patterns rather than intrinsic properties of the surgical approach alone. Definitions of recurrence varied widely, ranging from radiographic reaccumulation to strictly symptomatic thresholds, and imaging schedules were inconsistently reported, complicating temporal interpretation of failure mechanisms. Device-specific effects were further obscured in studies that aggregated multiple MIS platforms under a single category. Functional outcomes were infrequently reported, and frailty indices (critical determinants of both treatment selection and outcomes in elderly populations) were rarely incorporated. Finally, the absence of standardised reporting on postoperative evacuation completeness precluded quantitative assessment of immediate procedural success.

### Future directions

Future research must focus on the following. First, device-stratified RCTs comparing SEPS, twist-drill systems, YL-1, and BHC. Instead of treating MIS and BHC as monolithic categories, future work should compare specific devices and assess heterogeneity of treatment protocols, drainage strategies, and follow-up imaging. Second, mechanistic imaging studies evaluating brain re-expansion kinetics, residual membrane architecture, and pneumocephalus dynamics. Third, standardised definitions of recurrence, reoperation, and complications to harmonise global data. Fourth, stratified treatment algorithms integrating frailty, haematoma morphology, and membrane vascularity. Finally, economic modelling incorporating both procedural efficiency and recurrence-driven resource utilisation. Such studies are essential to move beyond descriptive comparisons toward physiologically-informed, precision surgical strategies.

## Conclusion

This systematic review and meta-analysis demonstrates that MIS offers meaningful perioperative advantages, including reduced OT and LOS, but is associated with higher reoperation risk compared with BHC, particularly for SEPS-based techniques. Despite these differences in durability, mortality and functional outcomes were similar between groups, though these findings should be interpreted cautiously given heterogeneity in outcome definition, timing of assessment, and limited reporting across studies. BHC therefore represents the more durable and reliable intervention for cSDH, while MIS retains a role in carefully selected patients, particularly when minimising operative burden is prioritised. While MIS offers procedural advantages, the overall balance of evidence favours BHC as the primary surgical strategy in most patients. MIS may be best considered a complementary strategy rather than an equivalent alternative. As adjunctive strategies such as MMAE continue to evolve, future treatment paradigms will need to integrate these dimensions to optimise outcomes in cSDH.

## Supplementary Information

Below is the link to the electronic supplementary material.

ESM 1 Supplementary Material 1 (DOCX 5.51 MB)

## Data Availability

No datasets were generated or analysed during the current study.
