# Supplementary material for: Minimally invasive versus burr hole craniostomy for chronic subdural haematoma evacuation: a systematic review and meta-analysis
Source: Acta Neurochir (Wien). 2026 May 22;168(1):171. doi: 10.1007/s00701-026-06912-5 (PMC13427998; doi:10.1007/s00701-026-06912-5)
Supplement: Supplementary file 1 — Supplementary Material 1 (DOCX 5.51 MB) [file 701_2026_6912_MOESM1_ESM.docx]

| **Database** | **Search Strategy** |
| --- | --- |
| PubMed  (n=201) | ( "Hematoma, Subdural, Chronic"[Mesh] OR "chronic subdural hematoma"[Title/Abstract] OR "CSDH"[Title/Abstract] OR "chronic subdural haematoma"[Title/Abstract] OR "subacute subdural hematoma"[Title/Abstract] )  AND  ("twist drill"[Title/Abstract] OR "twist-drill"[Title/Abstract] OR "TDC"[Title/Abstract] OR "hollow screw"[Title/Abstract] OR "SEPS"[Title/Abstract] OR "subdural evacuating port system"[Title/Abstract] OR "minimally invasive puncture"[Title/Abstract] OR "YL-1"[Title/Abstract] OR "bedside drainage"[Title/Abstract] OR "trephination"[Title/Abstract])  AND  ("Trephining"[Mesh] OR "burr-hole"[Title/Abstract] OR "burr hole"[Title/Abstract] OR "burrhole"[Title/Abstract] OR "BHC"[Title/Abstract] OR "craniostomy"[Title/Abstract]) |
| Embase  (n=398) | ('chronic subdural hematoma' or CSDH or 'chronic subdural haematoma' or 'subacute subdural hematoma').mp.  AND  ('twist drill' or 'twist-drill' or TDC or 'hollow screw' or SEPS or 'subdural evacuating port system' or 'minimally invasive puncture' or YL-1 or 'bedside drainage' or trephination).mp.  AND  ('trephination' or 'burr-hole' or 'burr hole' or burrhole or BHC or craniostomy).mp. |
| Medline  (n=181) | exp Hematoma, Subdural, Chronic/ or "chronic subdural hematoma".ti,ab. or CSDH.ti,ab. or "chronic subdural haematoma".ti,ab. or "subacute subdural hematoma".ti,ab.  AND  ("twist drill" or "twist-drill" or TDC or "hollow screw" or SEPS or "subdural evacuating port system" or "minimally invasive puncture" or YL-1 or "bedside drainage" or trephination).ti,ab.  AND  exp Trephining/ or "burr-hole".ti,ab. or "burr hole".ti,ab. or burrhole.ti,ab. or BHC.ti,ab. or craniostomy.ti,ab. |
| CENTRAL  (n=59) | ("chronic subdural hematoma" OR CSDH OR "chronic subdural haematoma" OR "subacute subdural hematoma")  AND  ("twist drill" OR "twist-drill" OR TDC OR "hollow screw" OR SEPS OR "subdural evacuating port system" OR "minimally invasive puncture" OR YL-1 OR "bedside drainage" OR trephination)  AND  ("burr-hole" OR "burr hole" OR burrhole OR BHC OR craniostomy OR trephination) |

**Supplementary Table 1.** Search Strategies, n = number of articles returned

| **No.** | **Item** | **Outcome** |
| --- | --- | --- |
| 1 | Did the research questions and inclusion criteria for the review include the components of PICO? | Y |
| 2 | *Did the report of the review contain an explicit statement that the review methods were established prior to the conduct of the review and did the report justify any significant deviations from the protocol? | Y |
| 3 | Did the review authors explain their selection of the study designs for inclusion in the review? | Y |
| 4 | *Did the review authors use a comprehensive literature search strategy? | Y |
| 5 | Did the review authors perform study selection in duplicate? | Y |
| 6 | Did the review authors perform data extraction in duplicate? | Y |
| 7 | *Did the review authors provide a list of excluded studies and justify the  exclusions? | Y |
| 8 | Did the review authors describe the included studies in adequate detail? | Y |
| 9 | *Did the review authors use a satisfactory technique for assessing the  risk of bias (RoB) in individual studies that were included in the review? | Y |
| 10 | Did the review authors report on the sources of funding for the studies  included in the review? | Y |
| 11 | *If meta-analysis was performed did the review authors use appropriate  methods for statistical combination of results? | Y |
| 12 | If meta-analysis was performed, did the review authors assess the potential impact of RoB in individual studies on the results of the meta-analysis or other evidence synthesis? | Y |
| 13 | *Did the review authors account for RoB in individual studies when interpreting/ discussing the results of the review? | Y |
| 14 | Did the review authors provide a satisfactory explanation for, and discussion of, any heterogeneity observed in the results of the review? | Y |
| 15 | *If they performed quantitative synthesis did the review authors carry out an adequate investigation of publication bias (small study bias) and discuss its likely impact on the results of the review? | Y |
| 16 | Did the review authors report any potential sources of conflict of interest, including any funding they received for conducting the review? | Y |

**Supplementary Table 2.** AMSTAR 2 criteria for the present review. *Critical weaknesses

*
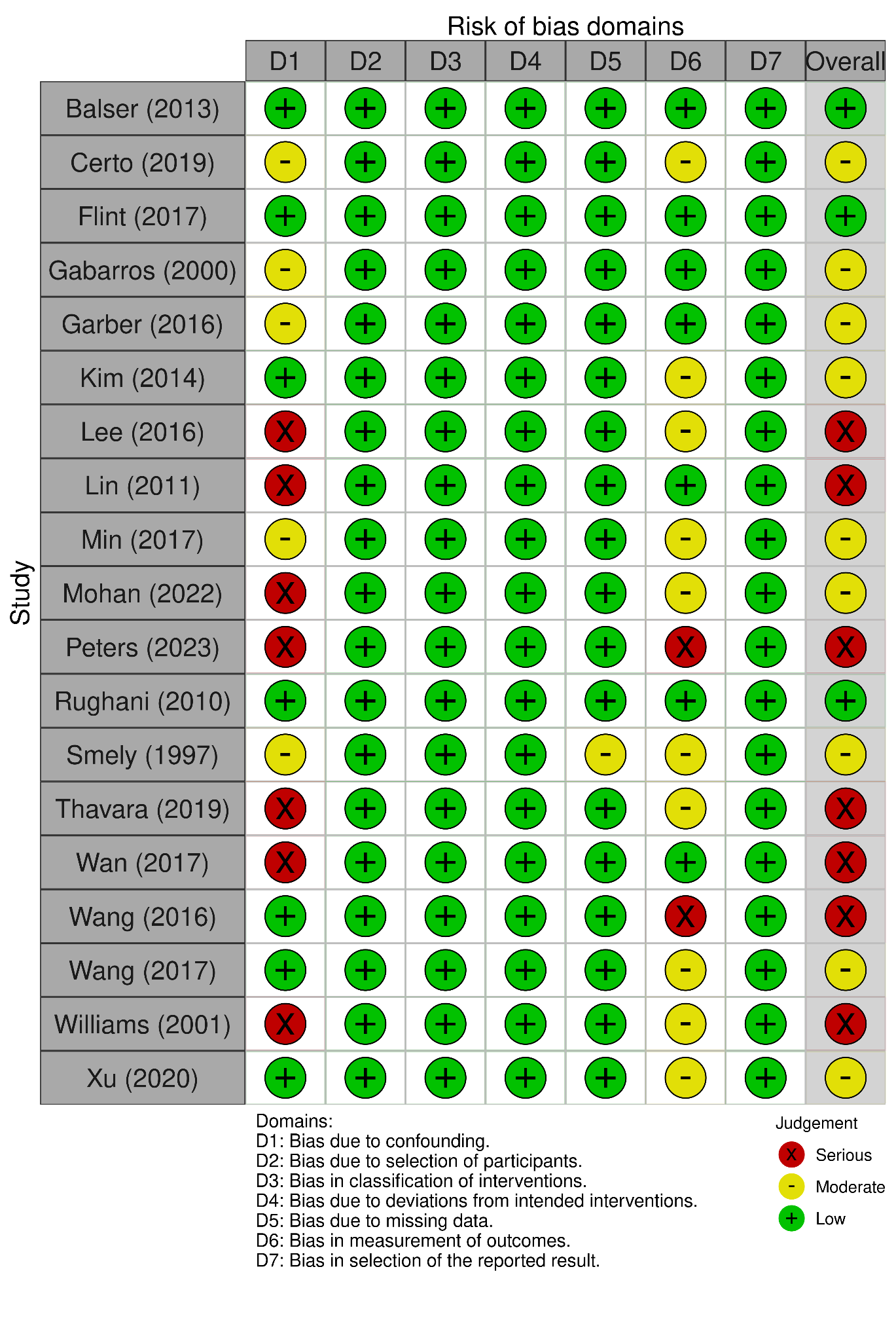
*

**Supplementary Table 3.** Risk of bias summary for non-randomized studies (ROBINS-I)


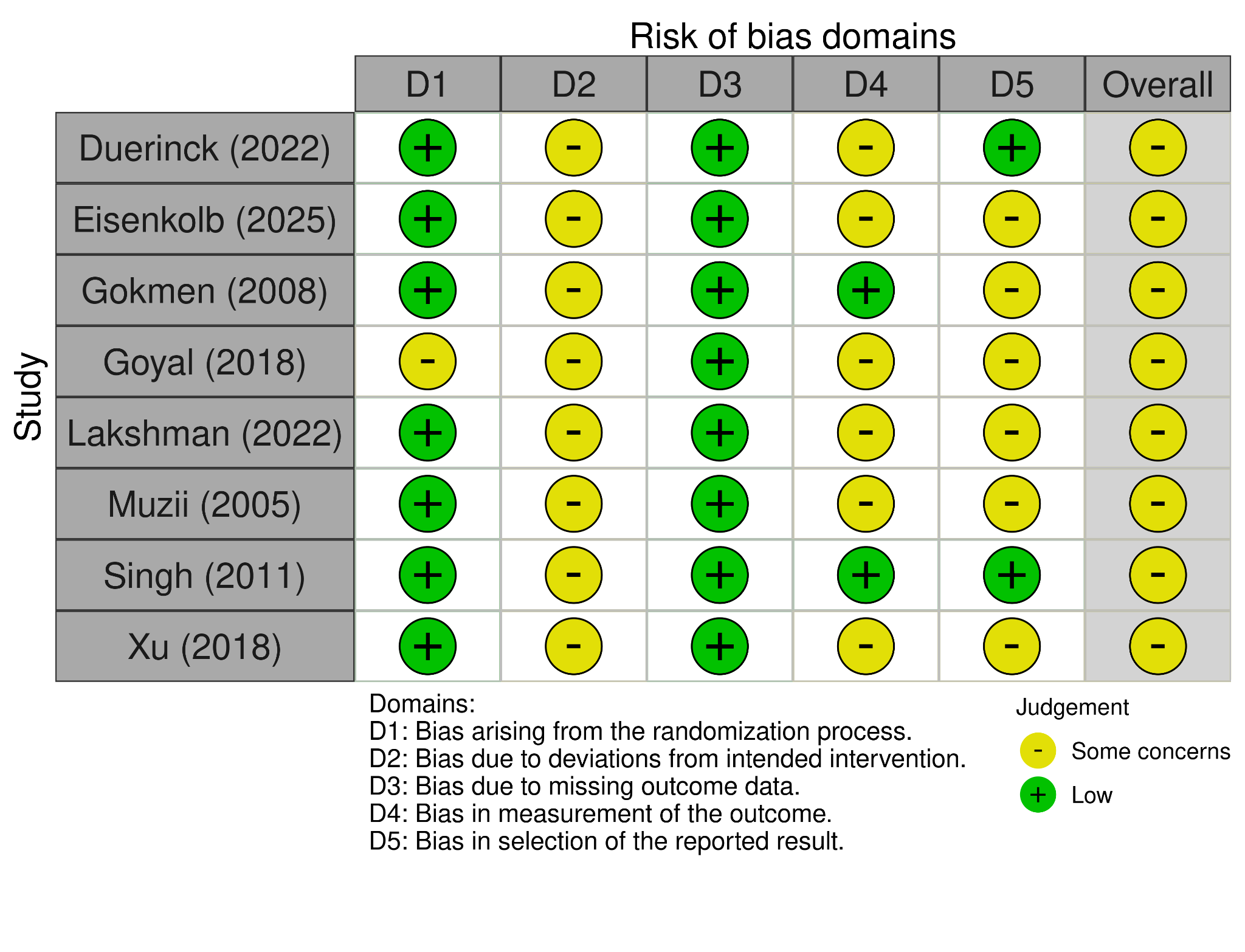


**Supplementary Table 4.** Risk of bias summary for randomized studies (RoB 2).


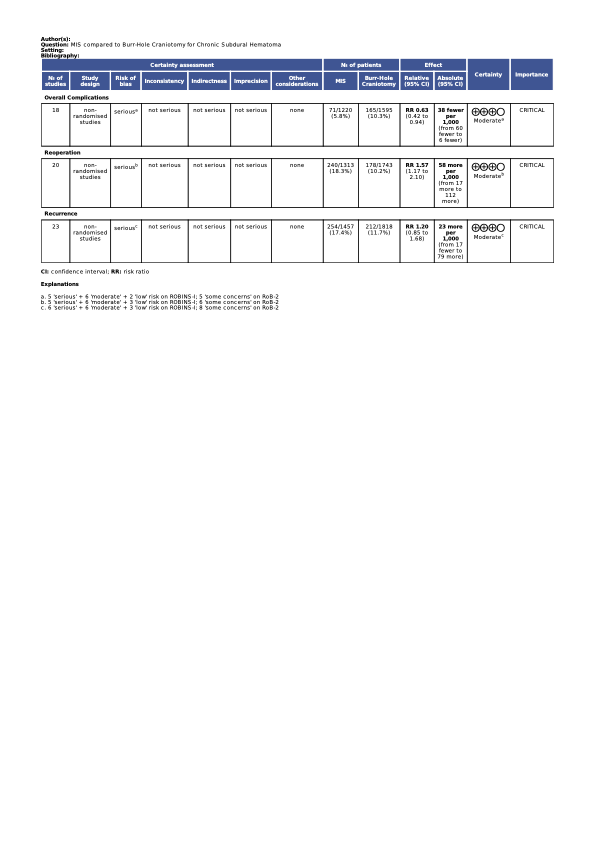


**Supplementary Table 5.** GRADE analysis.

| **Author** | **Critical Flaws** | **Non-Critical Flaws** | **Overall Confidence** |
| --- | --- | --- | --- |
| Liu et al., (2014) | **Item 2** - No explicit protocol registered before conducting the review  **Item 13** - Risk of bias scores not integrated into results or discussion | **Item 10** - Funding sources of included studies not reported  **Item 12** - Did not explore impact of RoB | **Critically Low** |
| Yagnik et al., (2021) | **Item 2** - No explicit protocol registered before conducting the review  **Item 7** - No list of excluded studies provided  **Item 9** - Risk of bias assessment inappropriate: used Newcastle-Ottawa Score (NOS) | **Item 6** - No clear statement that data extraction was performed in duplicate  **Item 10** - Funding sources of included studies not reported | **Critically Low** |
| Li et al., (2023) | **Item 2** - No explicit protocol registered before conducting the review  **Item 7** - No list of excluded studies provided  **Item 13** - Risk of bias scores not integrated into results or discussion | **Item 3** - No explicit justification for included study design.  **Item 10** - Funding sources of included studies not reported.  **Item 12** - Did not explore impact of RoB | **Critically Low** |
| Qiu et al., (2023) | **Item 13** - Risk of bias scores not integrated into results or discussion | **Item 10** - Funding sources of included studies not reported.  **Item 12** - Did not explore impact of RoB | **Critically Low** |
| Al-Salihi et al., (2023) | **Item 7** - No list of excluded studies provided | **Item 10** - Funding sources of included studies not reported.  **Item 12** - Did not explore impact of RoB | **Low** |
| Patel & Nischal et al., 2025 (this review) | **None** | **None** | **High** |

**Supplementary Table 6.** Summary of quality appraisal of this review compared to previous systematic reviews using AMSTAR-2 criteria.


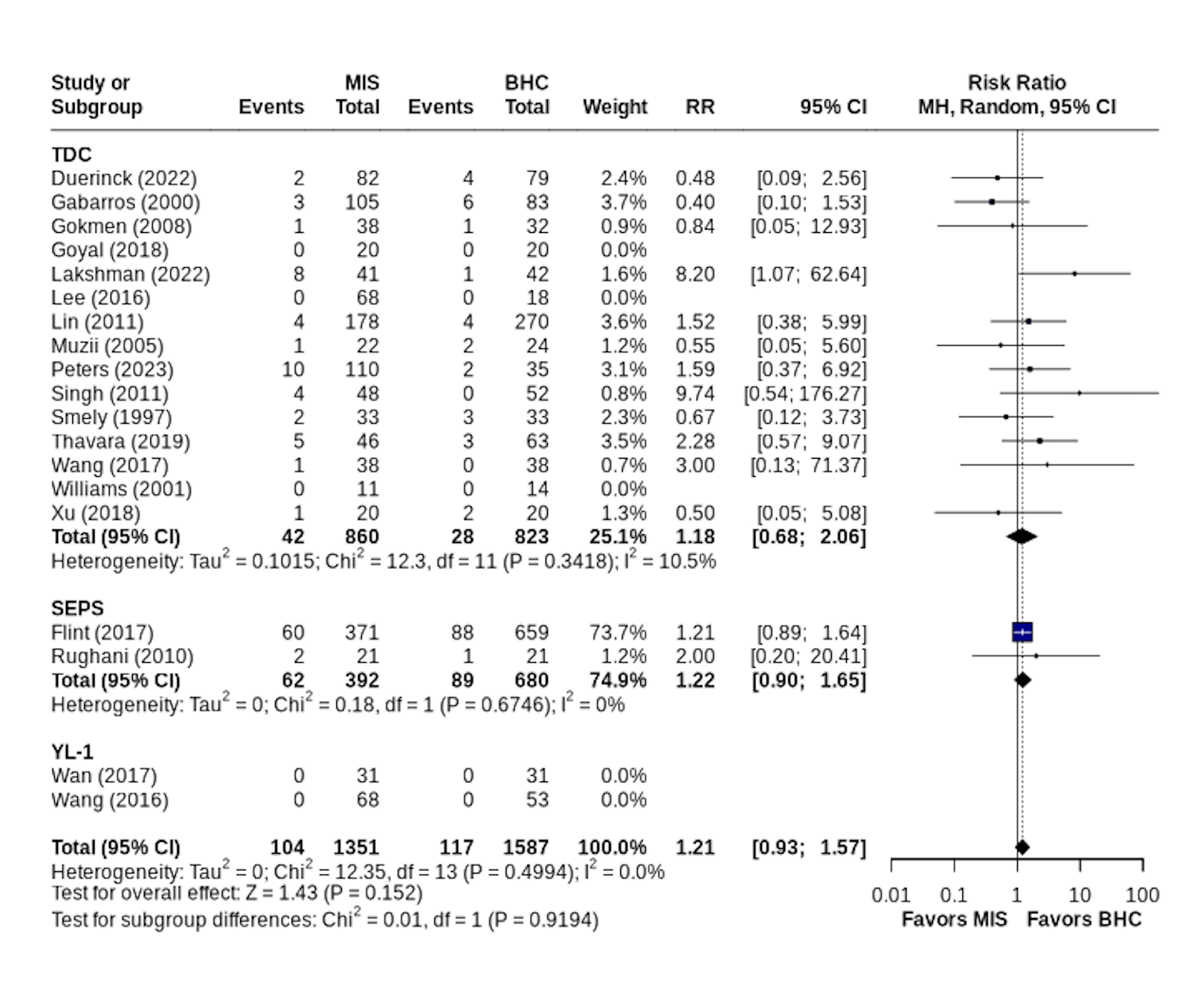


**Supplementary Figure 1.** Forest plot comparing mortality in patients following minimally invasive surgery (MIS) or burr hole craniostomy (BHC) with subgroup analysis by device type. Abbreviations: TDC: Twist Drill Craniostomy; SEPS: Subdural Evacuating Port System; YL-1: YL-1 Puncture Needle.


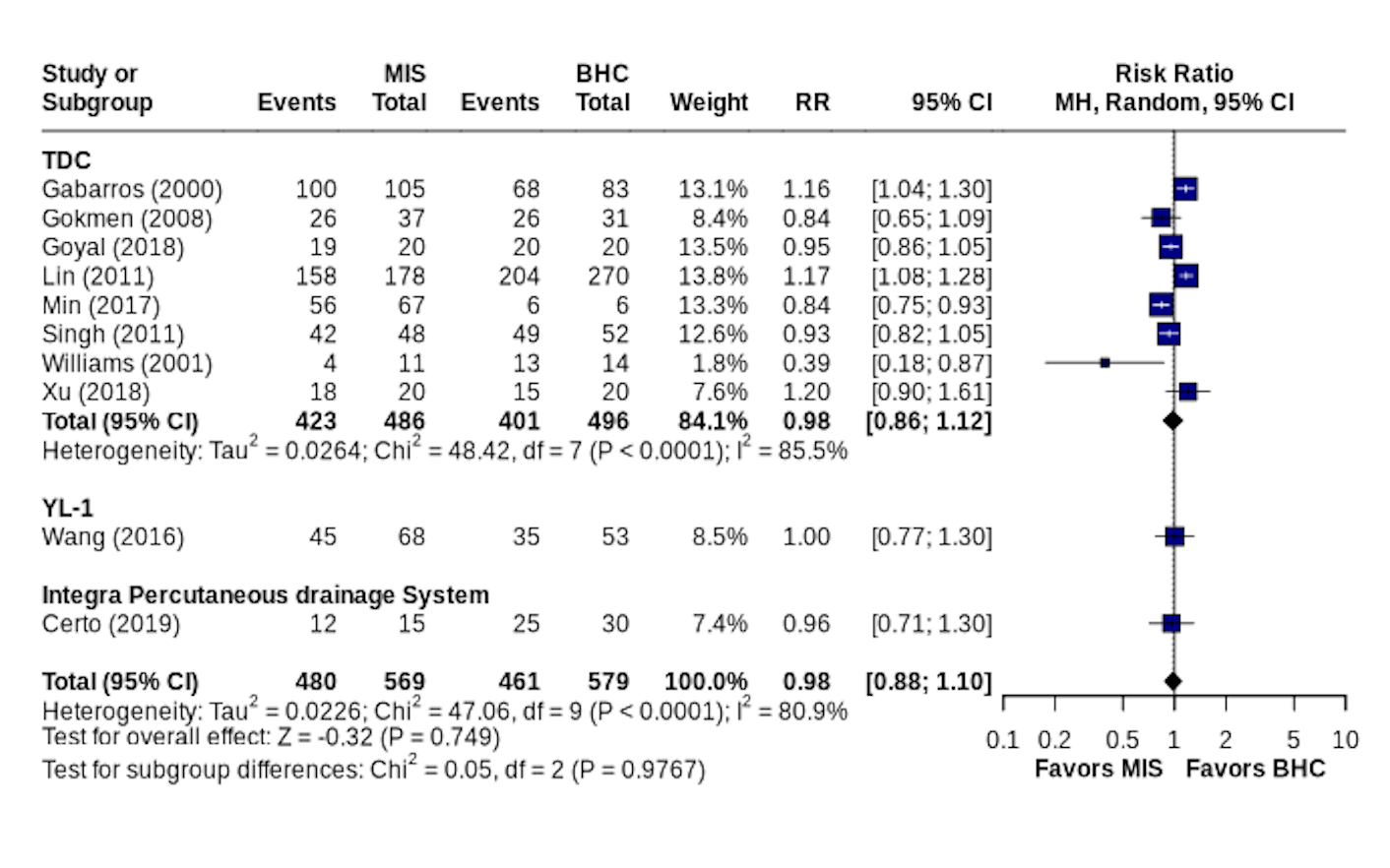


**Supplementary Figure 2.** Forest plot comparing complete clinical resolution in patients following minimally invasive surgery (MIS) or burr hole craniostomy (BHC) with subgroup analysis by device type. Abbreviations: TDC: Twist Drill Craniostomy; SEPS: Subdural Evacuating Port System; YL-1: YL-1 Puncture Needle.


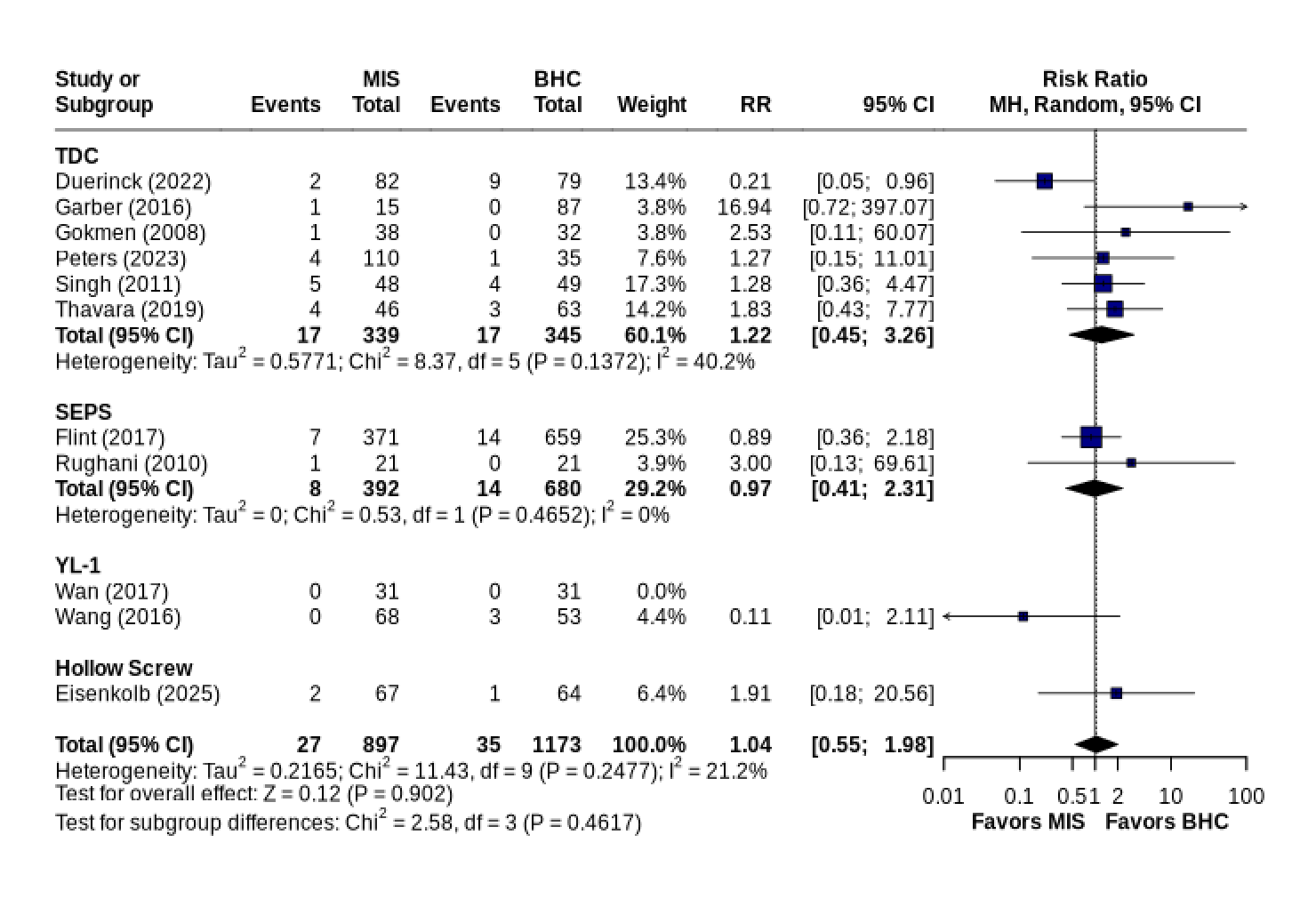


**Supplementary Figure 3.** Forest plot comparing bleeding in patients following minimally invasive surgery (MIS) or burr hole craniostomy (BHC) with subgroup analysis by device type. Abbreviations: TDC: Twist Drill Craniostomy; SEPS: Subdural Evacuating Port System; YL-1: YL-1 Puncture Needle.


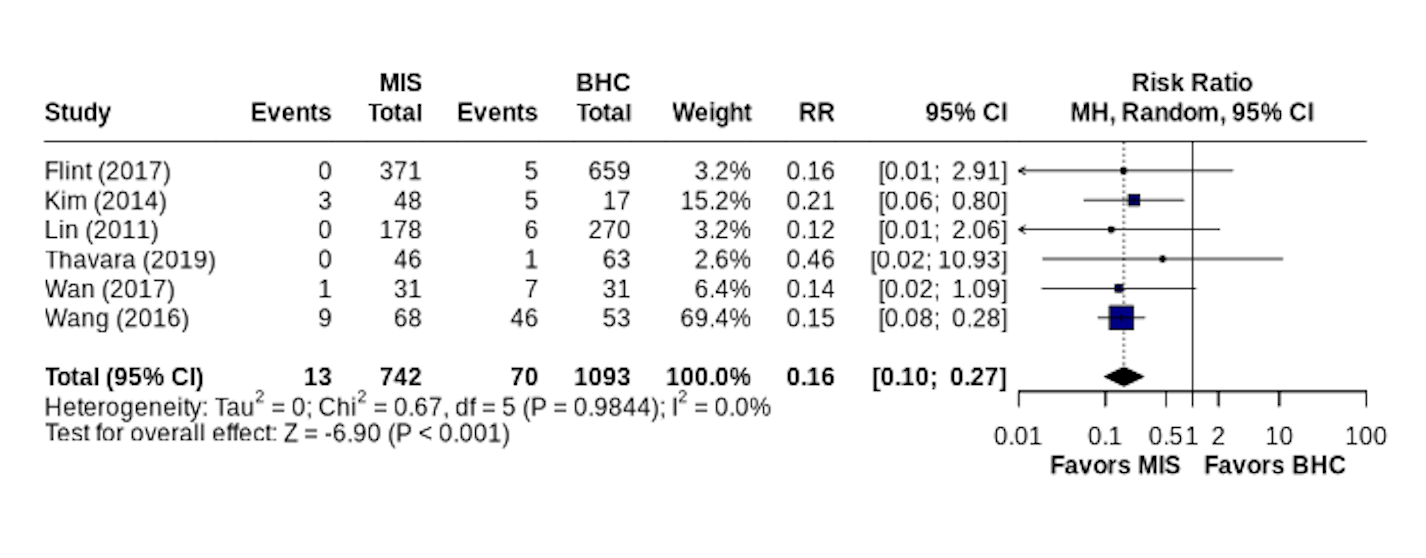


**Supplementary Figure 4.** Forest plot comparing pneumocephalus in patients following minimally invasive surgery (MIS) or burr hole craniostomy (BHC).


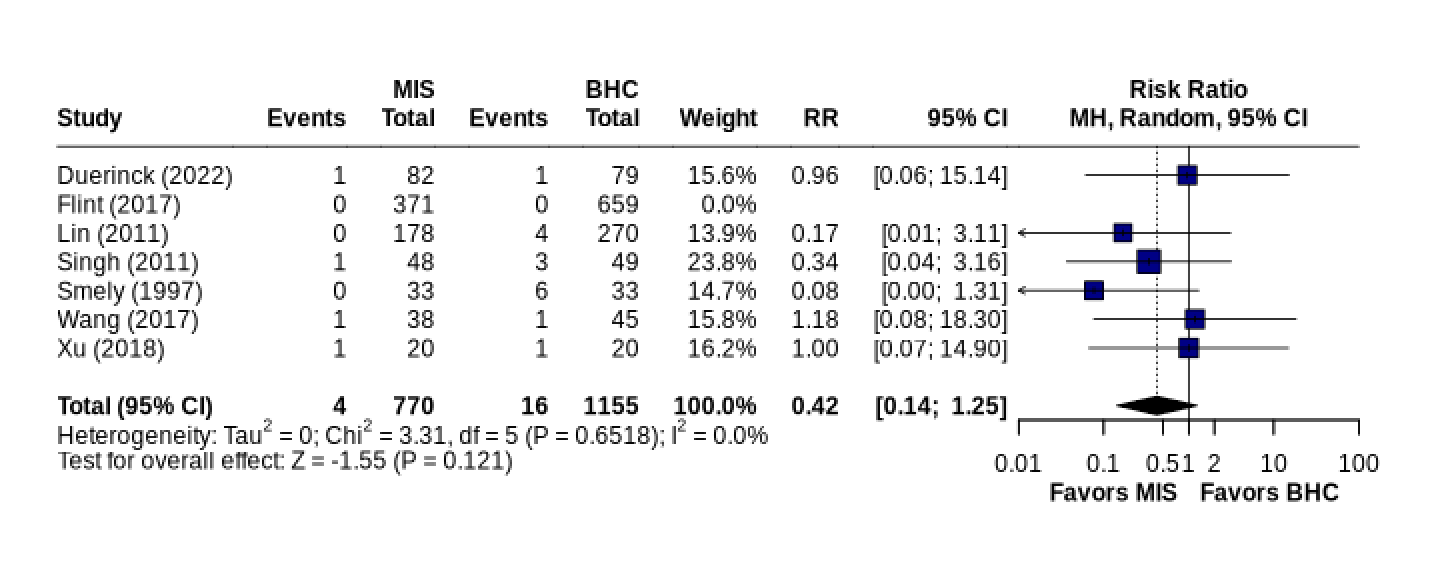


**Supplementary Figure 5.** Forest plot comparing wound infection in patients following minimally invasive surgery (MIS) or burr hole craniostomy (BHC).


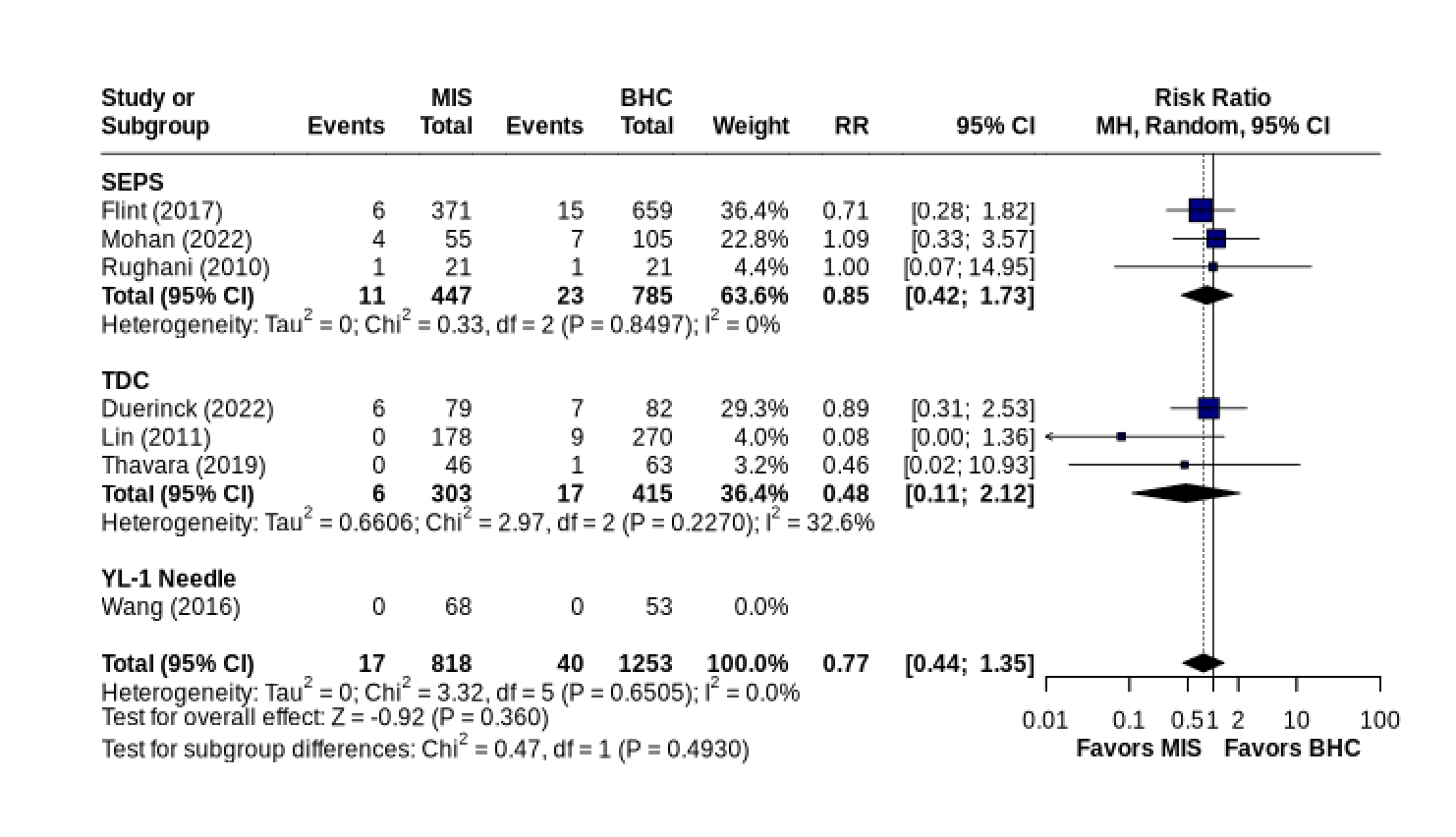


**Supplementary Figure 6.** Forest plot comparing seizure in patients following minimally invasive surgery (MIS) or burr hole craniostomy (BHC) with subgroup analysis by device type. Abbreviations: TDC: Twist Drill Craniostomy; SEPS: Subdural Evacuating Port System; YL-1: YL-1 Puncture Needle.


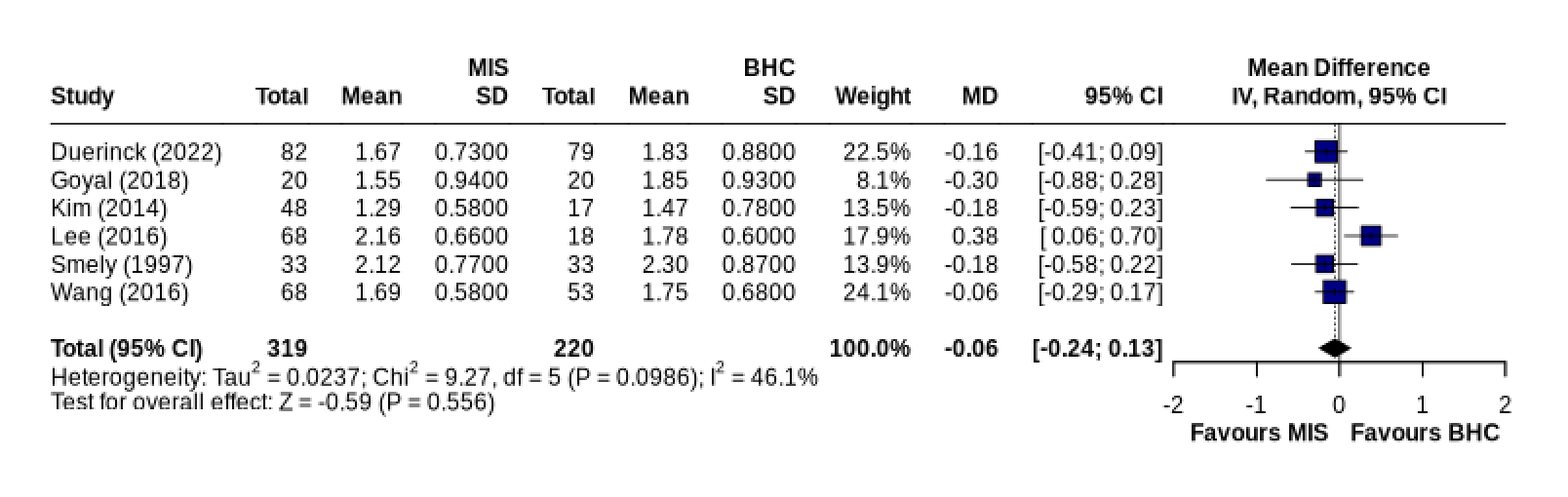


**Supplementary Figure 7.** Forest plot comparing pre-operative Markwalder Grading Scale (MGS) in patients following minimally invasive surgery (MIS) or burr hole craniostomy (BHC).


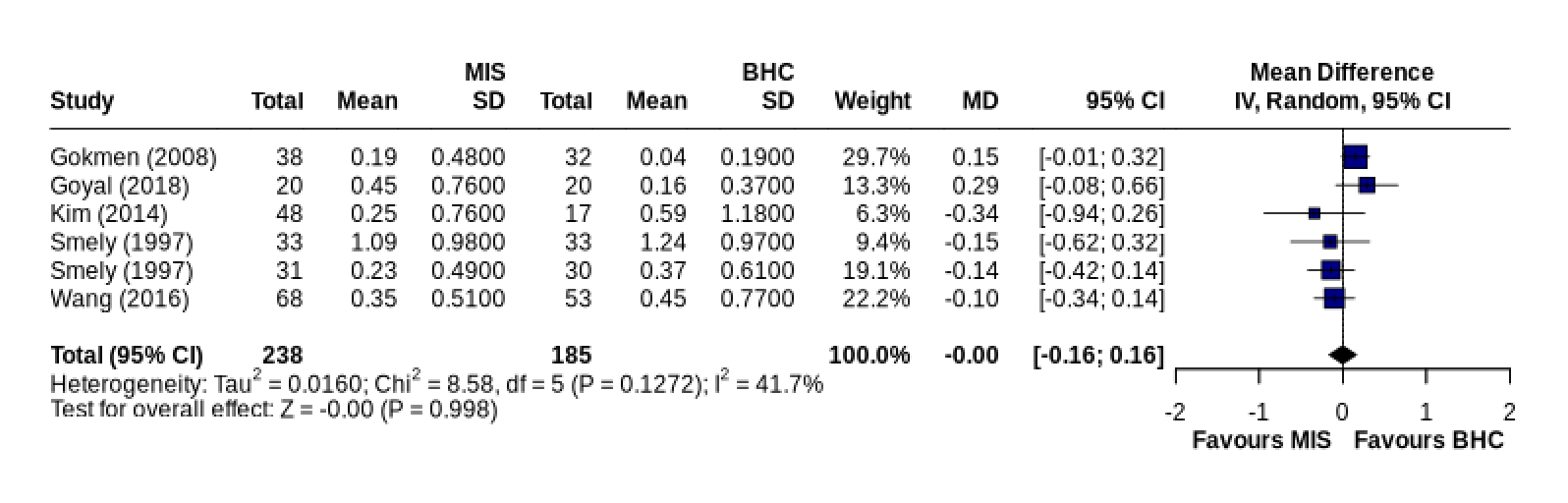


**Supplementary Figure 8.** Forest plot comparing post-operative Markwalder Grading Scale (MGS) in patients following minimally invasive surgery (MIS) or burr hole craniostomy (BHC).


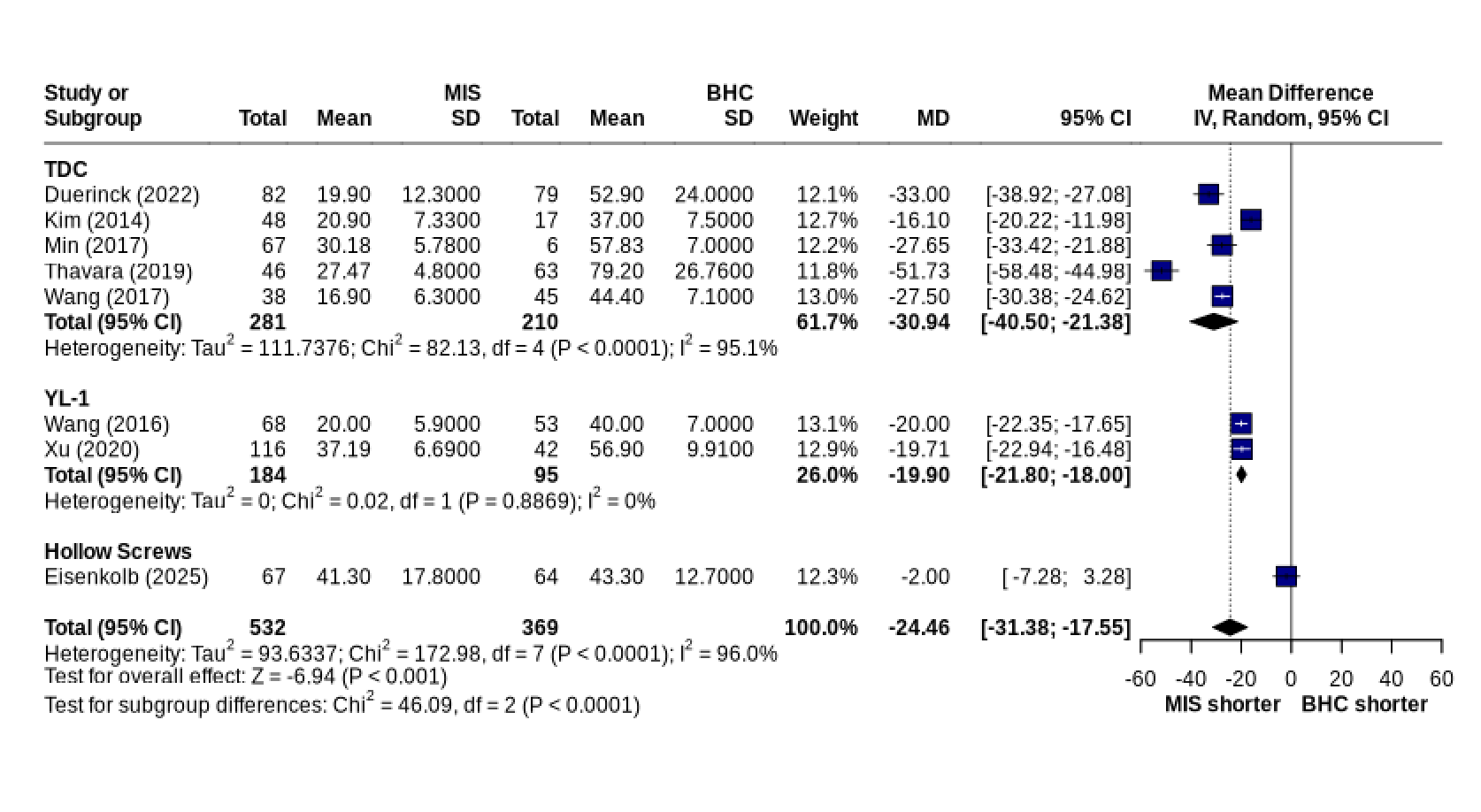


**Supplementary Figure 9.** Forest plot comparing operative time in patients following minimally invasive surgery (MIS) or burr hole craniostomy (BHC) with subgroup analysis by device type. Abbreviations: TDC: Twist Drill Craniostomy; YL-1: YL-1 Puncture Needle.


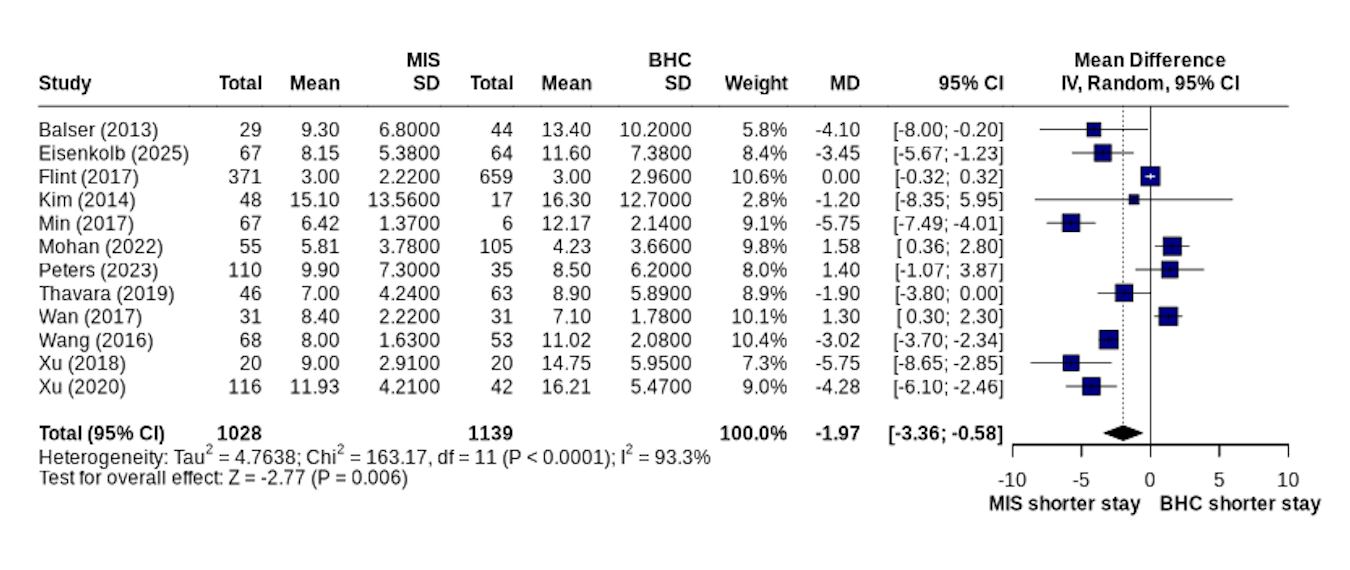


**Supplementary Figure 10.** Forest plot comparing length of stay in patients following minimally invasive surgery (MIS) or burr hole craniostomy (BHC).


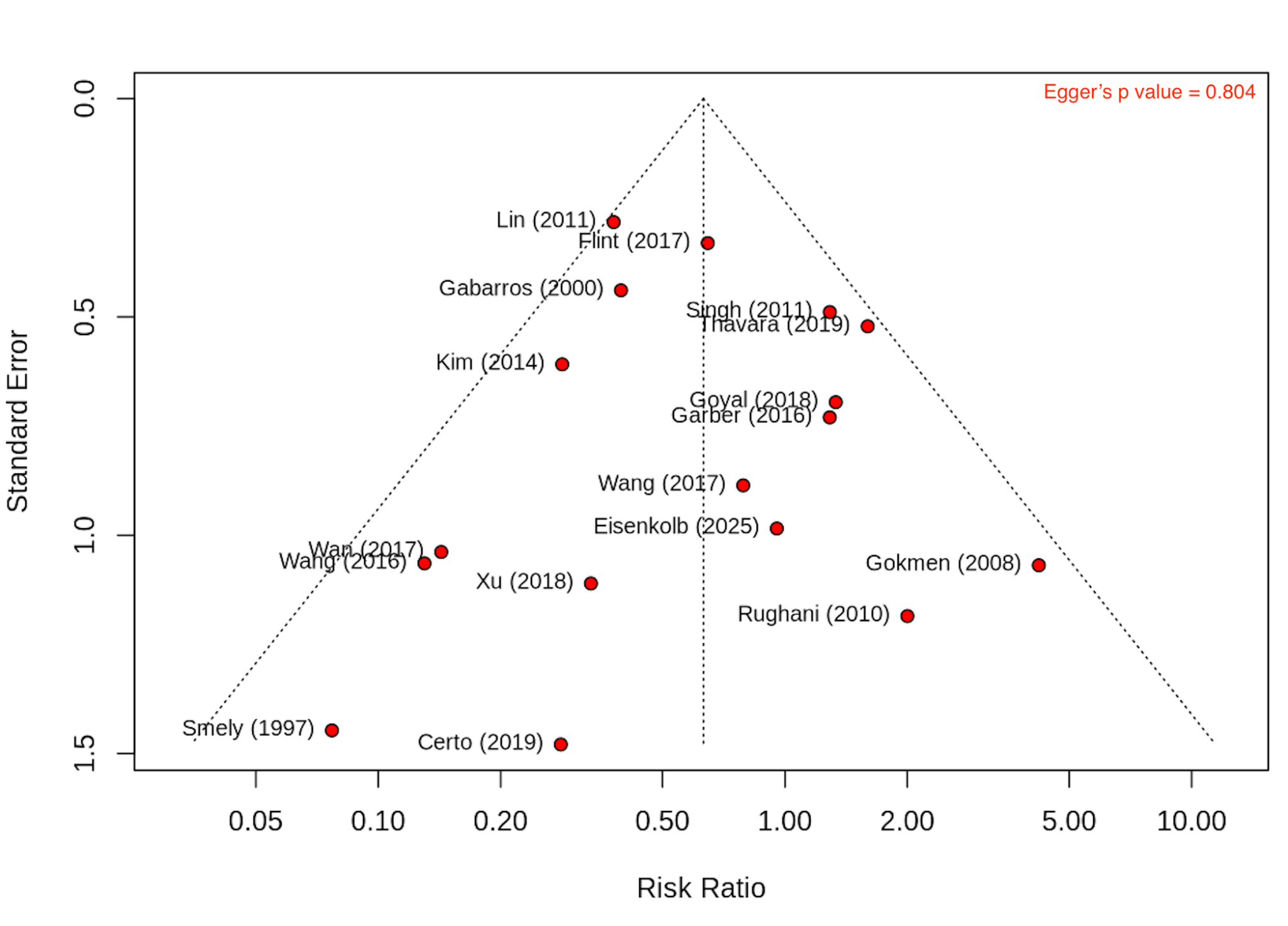


**Supplementary Figure 11.** Funnel plot for overall complications.
